# The topology, structure and PE interaction of LITAF underpin a Charcot-Marie-Tooth disease type 1C

**DOI:** 10.1186/s12915-016-0332-8

**Published:** 2016-12-07

**Authors:** Anita K. Ho, Jane L. Wagstaff, Paul T. Manna, Lena Wartosch, Seema Qamar, Elspeth F. Garman, Stefan M. V. Freund, Rhys C. Roberts

**Affiliations:** 1 Cambridge Institute for Medical Research, University of Cambridge, Cambridge Biomedical Campus, Cambridge, CB2 0XY UK; 2 MRC Laboratory of Molecular Biology, Francis Crick Avenue, Cambridge Biomedical Campus, Cambridge, CB2 0QH UK; 3 Department of Biochemistry, University of Oxford, South Parks Road, Oxford, OX1 3QU UK

**Keywords:** Lipopolysaccharide-induced tumour necrosis factor-α factor, Charcot-Marie-Tooth disease, Neuropathy, Endosomes

## Abstract

**Background:**

Mutations in Lipopolysaccharide-induced tumour necrosis factor-α factor (*LITAF*) cause the autosomal dominant inherited peripheral neuropathy, Charcot-Marie-Tooth disease type 1C (CMT1C). *LITAF* encodes a 17 kDa protein containing an N-terminal proline-rich region followed by an evolutionarily-conserved C-terminal ‘LITAF domain’, which contains all reported CMT1C-associated pathogenic mutations.

**Results:**

Here, we report the first structural characterisation of LITAF using biochemical, cell biological, biophysical and NMR spectroscopic approaches. Our structural model demonstrates that LITAF is a monotopic zinc-binding membrane protein that embeds into intracellular membranes via a predicted hydrophobic, in-plane, helical anchor located within the LITAF domain. We show that specific residues within the LITAF domain interact with phosphoethanolamine (PE) head groups, and that the introduction of the V144M CMT1C-associated pathogenic mutation leads to protein aggregation in the presence of PE.

**Conclusions:**

In addition to the structural characterisation of LITAF, these data lead us to propose that an aberrant LITAF-PE interaction on the surface of intracellular membranes contributes to the molecular pathogenesis that underlies this currently incurable disease.

**Electronic supplementary material:**

The online version of this article (doi:10.1186/s12915-016-0332-8) contains supplementary material, which is available to authorized users.

## Background

The Charcot-Marie-Tooth diseases (CMT) are among the most common inherited neurological disorders, with an estimated prevalence of 1 in 2500 [1]. CMT is associated with progressive degeneration of peripheral nerves, leading to muscle wasting and weakness, sensory deficits and limb deformities causing significant morbidity across populations [2].

Peripheral nerves are composed of two main functional anatomical structures: axons and Schwann cells. Axons convey electrical signals to and from the central nervous system and peripheries, while Schwann cells are neural crest-derived cells that engulf peripheral axons providing structural and trophic support. Furthermore, Schwann cells can also be stimulated to wrap multiple layers of plasma membrane around larger axons to form the insulating myelin sheath, a process known as myelination. Consistent with this anatomical and functional dichotomy, CMT can additionally be classified into ‘axonal’ and ‘demyelinating’ forms, reflecting the main sites of cellular dysfunction as the axon or Schwann cell, respectively [3].

With the advent of next-generation DNA sequencing, mutations in more than 80 genes have now been shown to be associated with CMT, highlighting key genes essential for peripheral nerve function [4]. Of the genes associated with demyelinating CMT, where Schwann cell dysfunction is thought to be the primary underlying pathology, the encoded proteins can be classified into three main groups: structural proteins of the myelin sheath, transcription factors that activate the myelination programme and, interestingly, proteins that are known or predicted to function in intracellular membrane traffic [5]. While Schwann cell-specific expression of CMT-associated proteins explains particular subtypes of demyelinating CMT [6, 7], most CMT-associated genes with known or predicted roles in membrane traffic are widely expressed across diverse tissues and species. Therefore, understanding why mutations in these widely expressed genes lead to isolated demyelinating peripheral neuropathies requires further study at the molecular level.

**Fig. 1:**
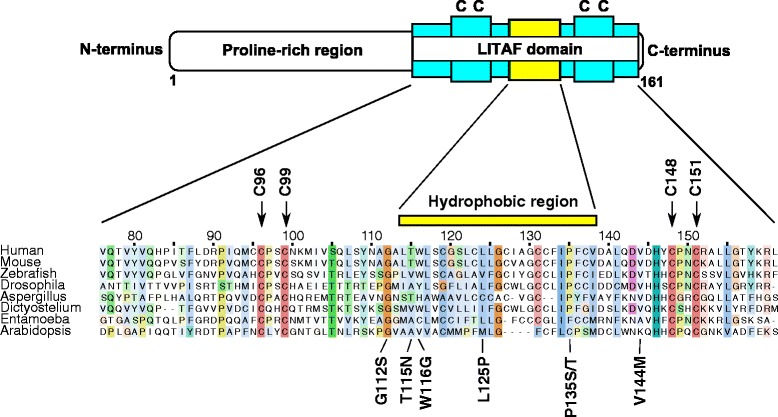
The domain organisation of LITAF. Schematic diagram to illustrate the domain organisation of the LITAF protein. The N-terminus is characterised by a proline-rich region which is followed by a LITAF domain at the C-terminus. The LITAF domain consists of two pairs of cysteine residues (indicated with *arrows* and coloured *red*) either side of a hydrophobic region. The amino acid sequences of selected LITAF domains taken from a variety of eukaryotes are shown to highlight the high degree of conservation of critical amino acid residues across species. Residues are numbered according to the human LITAF sequence and coloured according to the Clustalx scheme as implemented in the Jalview program to highlight conserved amino acid properties. Shading indicates the degree of conservation. The position of known CMT1C-associated pathogenic mutations are shown below the alignment.

CMT type 1C (CMT1C) is an autosomal dominant demyelinating peripheral neuropathy associated with mutations in *LITAF* (Lipopolysaccharide-induced tumour necrosis factor-α factor) [8]. *LITAF*, which is also known as *SIMPLE*, encodes a 17 kDa protein that targets endocytic structures and contains an N-terminal proline-rich region predicted to modulate protein-protein interactions, and a C-terminal region predicted to encode a ‘LITAF domain’ (sometimes also referred to as a ‘SIMPLE-like domain’) consisting of conserved cysteine residues separated by a 22 residue hydrophobic region (Fig. 1) [9–15]. LITAF domains are conserved in eukaryotes. Initial reports had proposed that LITAF functions as a transcription factor [16]; however, a consensus has now emerged that LITAF targets to intracellular membranes and is therefore likely to play a role in endocytic membrane trafficking [9–11, 13, 14, 17, 18]. Furthermore, LITAF has been shown to interact with proteins involved in receptor trafficking and degradation in tissue cell lines [9, 13, 19, 20]. Despite the evolutionary conservation and widespread tissue expression, no structural data currently exist concerning the LITAF domain. On the contrary, conflicting models regarding the topology and fold of this domain have been proposed, without supporting experimental data [11, 15, 17].

Here, we present the membrane topology, metal binding and structural features of the human LITAF protein. The biophysical and cell biological effects of the recruitment of interacting proteins to LITAF are then reported, demonstrating the importance of short peptide sequence motifs in the N-terminal proline-rich region for LITAF’s intracellular localisation. Finally, we show that distinct residues contained within the LITAF domain interact with phosphoethanolamine (PE) head groups and that the alteration of these interactions leads to protein aggregation in the presence of a pathogenic CMT1C mutation, providing molecular insights into the underlying pathogenesis of a subtype of this currently incurable, common, inherited disease.

## Results

### LITAF domains are evolutionarily conserved

LITAF domains are found throughout the eukaryotes, suggesting ancient conserved functions (Additional file 1: Figure S1), with multiple instances of expansion, especially in the metazoa. Of relevance to the current study, one particular duplication appears to have occurred in the common ancestor of the jawed vertebrates (gnathostomes), leading to the eventual existence of two LITAF domain-containing genes in humans: the so-far uncharacterised *CDIP1* gene, and the CMT1C-associated gene, *LITAF* (Additional file 2: Figure S2). Intriguingly, and despite the large lineage-specific expansions seen in the LITAF domain gene family as described, the canonical domain architecture – that is, a proline-rich domain at the N-terminus followed by a C-terminal LITAF domain – is conserved across eukaryotes (with the exception of the apicomplexans), highlighting the likely requirement for both regions to be present for correct intracellular protein function (Additional file 3: Figure S3 and Additional file 4: Figure S4a).

With the aim of identifying key conserved features that are likely to be of critical functional importance, we gathered a cohort of broadly representative LITAF domain sequences from which a comprehensive sequence alignment was derived. This alignment revealed that two pairs of cysteines, located either side of a predicted hydrophobic helix with amphipathic properties, are strictly conserved (Fig. 1 and Additional file 4: Figure S4b, c). These cysteine pairs, in the absence of the intervening helix, are reminiscent of metal-ion coordinating residues found in zinc-finger-like structures [21]. Six additional cysteine residues are present in the predicted hydrophobic helical region of the human LITAF domain, but are less well conserved across our alignment. As their sequence positions are not consistent with the typical known cysteine-containing metal coordinating motifs (e.g. CxxC/HxxC), it appears unlikely that these additional cysteine residues are involved in coordinating a metal ion.

Given that the luminal, transmembrane and cytosolic domains of proteins are conserved to a different extent in evolution [22, 23], the absolute conservation of these residues in tandem led us to hypothesise that the N- and C-termini of the LITAF domain are very unlikely to be separated by a phospholipid bilayer-traversing transmembrane domain as has been previously postulated [11]. Furthermore, most CMT1C-associated pathogenic mutations fall at conserved residues in this pan-eukaryotic alignment of LITAF domains, again highlighting key residues likely to play critical roles in maintaining the function of this ancient domain.

With these points in mind, we set out to re-examine the role of the LITAF domain in targeting the protein to membranes and to experimentally characterise the topology of the human LITAF protein.

### LITAF targets to membranes via the hydrophobic helical region

**Fig. 2:**
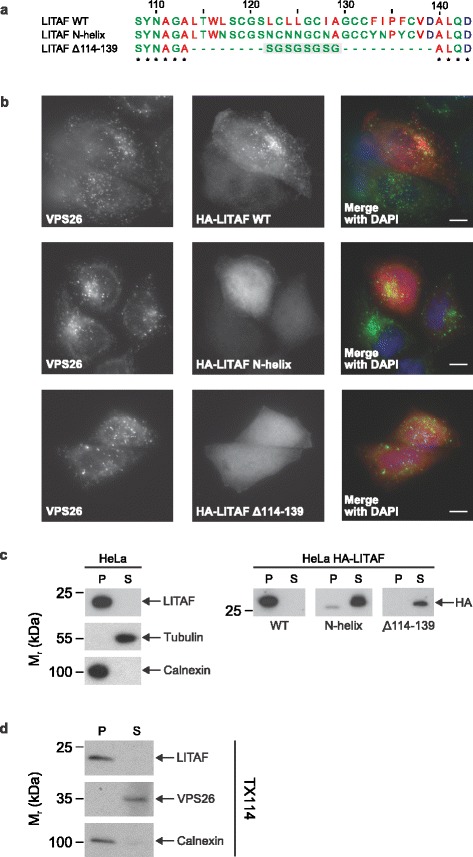
LITAF is anchored to membranes via hydrophobic residues contained within the LITAF domain. **a** Amino acid sequences of the hydrophobic regions present in the protein constructs used to determine membrane insertion. Residues are numbered according to the full length human LITAF sequence. Hydrophobic residues are coloured red, while acidic residues are coloured blue. Residues retained in all three constructs are indicated by asterisks below the sequence. The grey box denotes the serine–glycine linker added in place of the predicted helix in the Δ114–139 construct. **b** The HA-tagged LITAF constructs were transiently expressed in HeLa cells and the localisation determined by immunofluorescence microscopy. The endosomal targeting of endogenously expressed VPS26 is shown in the left panels for comparison. The middle panels show that the endosomal membrane targeting of LITAF is dependent on key hydrophobic residues within the hydrophobic region. Scale bar denotes 20 μm. **c** Membrane fractionation from HeLa cells expressing endogenous LITAF and transiently expressed HA-tagged LITAF constructs. While both endogenous and HA-tagged wild type LITAF is found in the membrane pellets (P), the LITAF constructs harbouring hydrophilic mutations (N-helix) and a deletion in the vicinity of the hydrophobic region (Δ114–139) are found in the soluble fraction (S). Calnexin and tubulin were used as integral membrane and soluble protein controls, respectively. **d** Wild type LITAF displays the characteristics of an integral membrane protein as determined by the extraction of the endogenous protein from HeLa cells using TX114. The integral membrane protein, Calnexin, and VPS26, which associates with, but does not insert into membranes, were used as controls

The targeting of LITAF to membranes has previously been shown to be via the C-terminal LITAF domain [11]. Consistent with previous reports, we found that LITAF predominantly targeted endocytic vesicles, colocalising with marker proteins of both early and late endosomes (Additional file 5: Figure S5). This endosomal targeting and association with membrane can be prevented by either deleting the predicted hydrophobic helical region within the LITAF domain (HA-LITAF Δ114–139) or by mutating eight hydrophobic residues contained therein (HA-LITAF N-helix) (Fig. 2a–c). These eight hydrophobic residues mutated in the N-helix construct are found on one side of the predicted helix according to our helical wheel analysis, consistent with an amphipathic character (Additional file 4: Figure S4c, d). By mutating only eight hydrophobic residues to arginine in generating the N-helix construct, the predicted helical nature of this region is preserved, leading us to hypothesise that, while the protein is rendered more soluble, the overall folding and architecture of the expressed molecule is maintained and remains similar to wild type. Endogenously expressed LITAF also possesses the biochemical properties of an integral membrane protein, as shown by the differential extraction of the protein with the detergent TX114 (Fig. 2d). Thus, our data suggest that LITAF is targeted and anchored to membranes via pivotal hydrophobic residues found within the predicted central hydrophobic helical region of the C-terminal LITAF domain.

### Both the N- and C-termini of LITAF are found on the cytosolic surfaces of vesicles

By analysing the sequences of LITAF domains from *Drosophila*, Ponting et al. [15] first postulated that the predicted hydrophobic helical region probably inserted into, but did not traverse, membranes. However, Lee et al. [11] later assumed that this hydrophobic region within the LITAF domain was a transmembrane helix, thereby concluding that LITAF must be C-terminally inserted into membranes. Importantly, neither hypothesis was supported by experimental data. We therefore used two complementary experimental approaches to determine the membrane topology of LITAF.

**Fig. 3:**
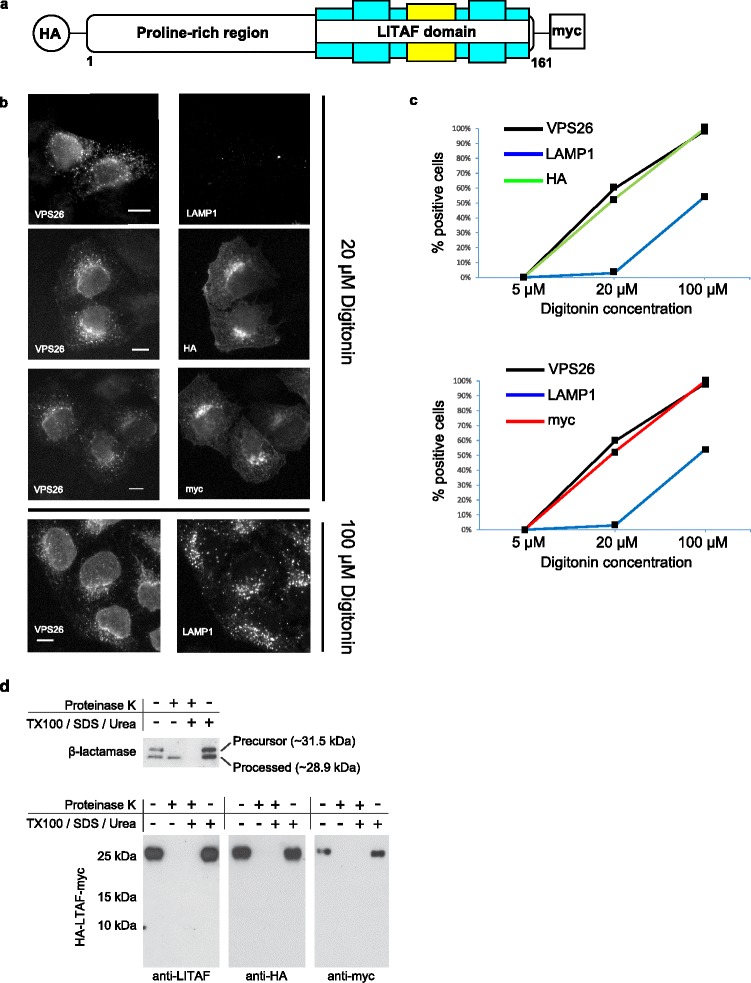
Membrane topology determination of LITAF. **a** Schematic diagram illustrating the LITAF protein tagged with HA at the N-terminus and myc at the C-terminus used in the experiments to determine the membrane topology. **b** Selective permeabilisation of the plasma membranes of HeLa cells stably expressing HA-LITAF-myc was achieved using 20 μM digitonin. Immunofluorescence microscopy was performed following incubation of the digitonin-permeabilised cells with antibodies towards HA and myc in addition to the control proteins, VPS26 and LAMP1, located on the cytosolic and luminal sides of endosomal membranes, respectively. The representative images show that both the N-terminal HA and C-terminal myc epitopes were detected concurrently with VPS26, indicating that both termini of LITAF are located on the cytosolic surface of membranes. In contrast, LAMP1 was not detected under these conditions. Permeabilsation of both plasma membrane and endosomal membranes was achieved at higher concentrations of digitonin as seen in the lower panel. **c** Representative graphs of a selective permeabilisation experiment illustrating the proportion of cells staining positive for HA (*top panel*) and myc (*bottom panel*) compared to VSP26 and LAMP1 (co-labelling and counting 400 cells) at increasing concentrations of digitonin. Note that all cells staining positive for HA were also positive for myc and that even at 100 μM digitonin, not all cells stained positive for LAMP1, indicating incomplete permeabilisation of endocytic vesicle membranes under these conditions. **d** HA-LITAF-myc was expressed using a rabbit reticulocyte lysate expression system in the presence of microsomal membranes to allow membrane insertion. A protease protection assay was then performed using proteinase K to determine the topology of the LITAF protein. In the presence of proteinase K, both N-terminal HA and C-terminal myc tags were digested, indicating that these epitopes are present on the surface of microsomal membranes (*bottom panel*). β-lactamase, containing a signal peptide that allows the translocation of a processed form to the interior of microsomal membranes, and hence protection from proteinase K digestion, was used as a control (*top panel*)

Firstly, we used a cellular semi-permeabilisation assay to determine whether the N- and C-termini of LITAF were located on the cytosolic or luminal membrane surfaces of endosomes, respectively [24]. To do this, we generated HeLa cells stably expressing LITAF epitope-tagged with HA on the N-terminus and myc on the C-terminus (Fig. 3a). Following selective permeabilisation of plasma membranes with 20 μM digitonin, both N-terminal HA and C-terminal myc epitope tags were detected by indirect immunofluorescence, concurrently with the cytosolic surface endosomal marker protein VPS26. In contrast, the LAMP1 epitope, found on the luminal side of endosomes and lysosomes, was not detected, confirming the maintained integrity of endocytic compartment membranes under these conditions (Fig. 3b, c). On the contrary, higher concentrations of digitonin (100 μM) permeabilised both plasma membranes and intracellular organelles/vesicles, exposing epitopes found on both the cytosolic and luminal surfaces of endosomes. These data are consistent with the hypothesis that the hydrophobic region of the LITAF domain does not traverse membranes, meaning that both the N- and C-termini face the cytosol.

Secondly, to verify our findings from the cellular semi-permeabilisation assay, we used a rabbit reticulocyte lysate cell-free system to express HA-LITAF-myc in the presence of microsomes [25, 26]. Once incorporated into microsomal membranes, the susceptibility of both the N- and C-terminal HA and myc epitope tags to protease digestion was ascertained. Using the protection of the processed form of β-lactamase within the microsomal lumen as control, we observed that both the HA and myc tags of LITAF were degraded following incubation with proteinase K (Fig. 3d). These data are therefore consistent with the semi-permeabilisation assay, and are strongly supportive of the hypothesis that both the N- and C- termini of LITAF are found on the cytosolic surface of membranes, and that the LITAF domain is embedded into membranes via the predicted hydrophobic helical region adopting an in-plane membrane anchor configuration [27].

### The LITAF domain coordinates zinc

**Fig. 4:**
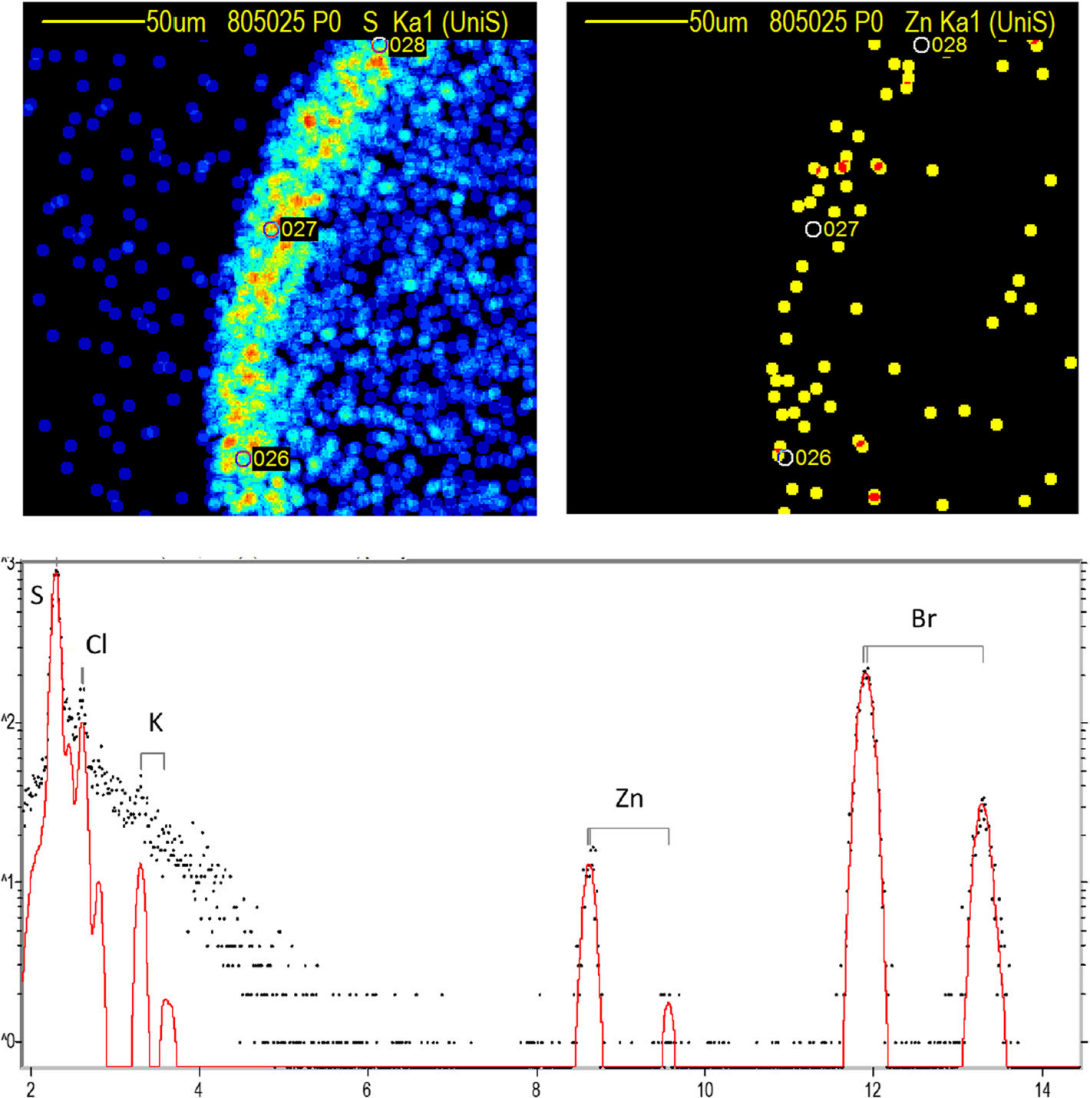
MicroPIXE analysis of LITAF Δ114–139. Top panels: Elemental maps (50 μm^2^) of the sulfur and zinc content of the LITAF Δ114–139 sample obtained from the proton induced X-ray emission (PIXE). The protein dries to the outside of the drop, giving an arc of sulfur which correlates with the zinc signal. The positions for the three-point spectra collected are marked. Bottom panel: X-ray energy spectrum for one of the points, showing the sulfur and zinc in the protein and the bromine from the buffer. Note the logarithmic scale on the *y*-axis

With the two well-conserved cysteine-containing ‘knuckles’ either side of the hydrophobic helical anchor (Fig. 1 and Additional file 4: Figure S4), reminiscent of intracellular zinc-binding motifs [21], we next asked whether LITAF is indeed a zinc-interacting protein. To address this question unequivocally, we performed microPIXE (proton-induced X-ray emission) analysis to determine the metal binding characteristics of LITAF protein constructs expressed and purified from *E. coli*. These experiments showed that wild type LITAF, LITAF N-helix and LITAF Δ114–139 contained 1.05 ± 0.21 [1.41, 1.05, 0.998], 1.55 ± 0.12 [1.42, 1.71, 1.51] and 0.90 ± 0.11 [0.86, 0.80, 1.05] zinc atoms/protein molecule (mean ± SD, n = 3, different points collected from the same sample of each protein), respectively (Fig. 4). Notably, LITAF constructs where one or more of the conserved cysteine residues had been replaced with alanine were all insoluble when expressed in *E. coli*, and also appeared as large puncta and could not be solubilised with TX100 when transiently expressed in HeLa cells, consistent with them being proteins prone to aggregation (Additional file 6: Figure S6). When not playing a direct functional or catalytic role, metal ions (most commonly zinc) often stabilise very small protein domains, enabling them to interact with other proteins or biomolecules [28]. These data therefore strongly suggest that the correct folding of the LITAF domain is dependent on the coordination of a zinc atom.

### The LITAF domain consists of cysteine-containing β-hairpins either side of the predicted hydrophobic helical anchor

We next set out to determine the structural characteristics of LITAF. The first NMR experiments of wild type LITAF in an extensive range of membrane-mimicking detergent environments highlighted the crucial importance of the correct choice of solubilising conditions. Under all conditions tested, we observed no signals that we would associate with a structured protein domain. Based on the assumption that these structured residues would be in close association with the membrane, and the high tendency of these samples to form oligomers and aggregate (Additional file 7: Figure S7), we refocused on the LITAF Δ114–139 construct, where the hydrophobic anchor had been removed, thereby rendering this construct soluble in the absence of detergents. ^13^C/^15^N-labelled LITAF Δ114–139 protein was straightforwardly expressed and purified from *E. coli* with an estimated molecular weight of 17 kDa, as determined using a 1D proton transverse relaxation experiment, consistent with a monomeric species in solution.

**Fig. 5:**
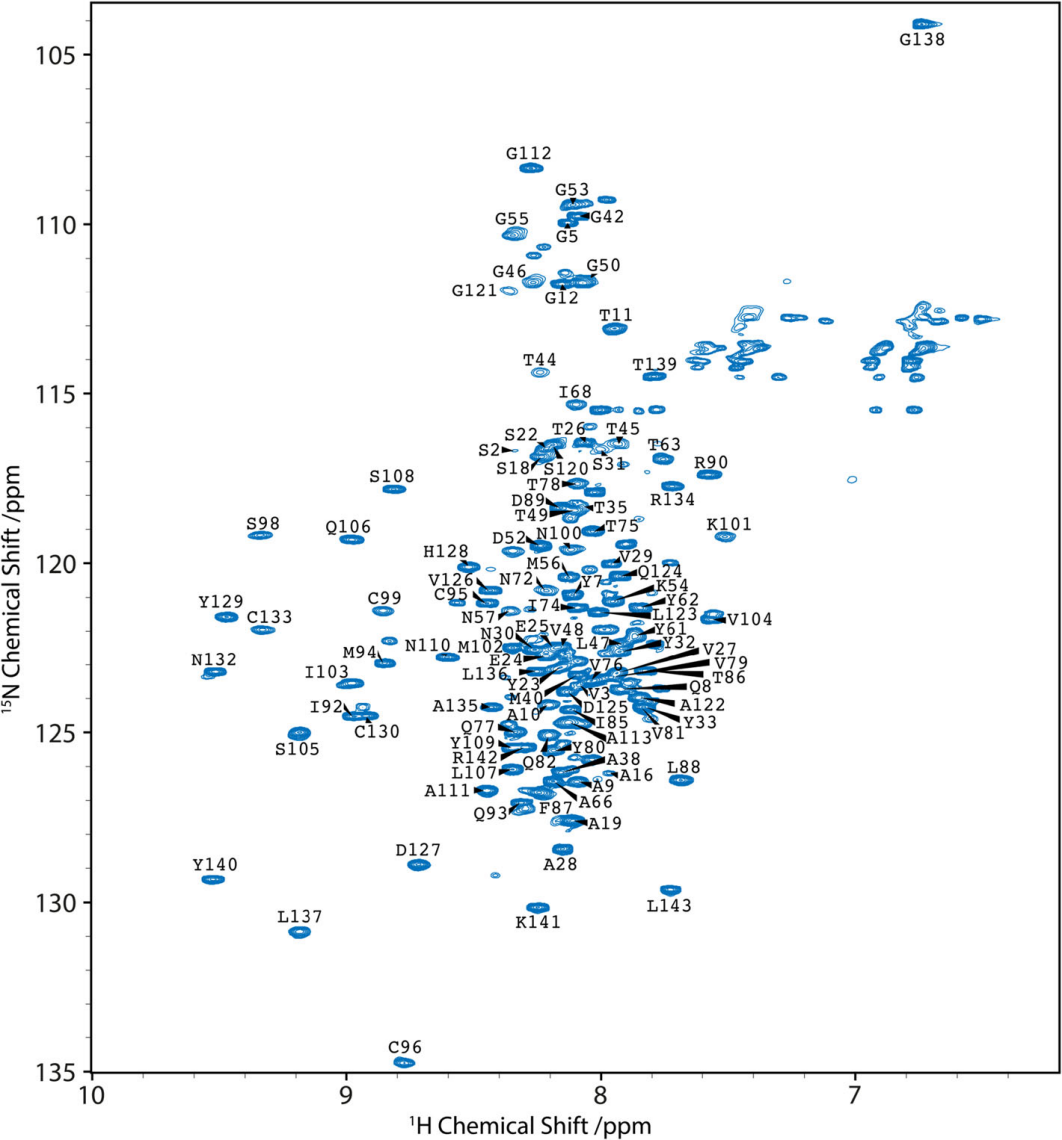
Assignment of the ^15^N BEST-TROSY of the LITAF Δ114–139 construct. Cross-peaks assigned to the structured LITAF domain have a wide proton distribution (~7.5 to 9.6 ppm) consistent with the secondary structure of β-strands. In contrast, cross-peaks assigned to the unstructured N-terminal proline-rich arm occupy the narrower random coil central region of the spectrum (7.8 to 8.4 ppm)

Initially, we performed standard NMR triple resonance backbone experiments to assign and analyse the individual amino acid residues within the LITAF Δ114–139 protein. The BEST-TROSY experiments focus on the backbone NH bonds of the protein, with each cross peak displayed corresponding to a specific assigned amino acid (Fig. 5). As mentioned above, the N-terminal region of LITAF contains multiple proline residues (which lack a backbone NH bond), and therefore the assignment of these amino acids also required the use of supplementary experiments (as outlined in Methods). Using these approaches, and despite missing assignments for the glycine-serine linker that replaces the hydrophobic region in the LITAF Δ114–139 construct, an 89% assignment (including the assignment of 20 out of 23 proline residues) was achieved.

Unsurprisingly, the LITAF N-helix construct also remained soluble in the absence of detergent. However, similarly to wild type LITAF, the assignment experiments on this construct were also unsuccessful, likely due to the tendency for the N-helix construct to form higher molecular weight oligomers (Additional file 7: Figure S7). Nevertheless, considerable overlap was evident between the N-helix and Δ114–139 constructs in the BEST-TROSY experiments (Additional file 8: Figure S8), suggesting that both LITAF constructs contain very similar secondary structure elements, implying that further interpretation of the Δ114–139 construct would enable key structural motifs of the wild type LITAF protein to be defined.

Building on the near complete assignment of the protein, we proceeded to determine which secondary structural elements are present in the LITAF Δ114–139 construct. This particular approach exploits the exquisite sensitivity of the backbone amino acid chemical shifts to their related secondary structure environment. By using the chemical shift analysis program, TALOS+ [29], we established that the LITAF domain of the Δ114–139 construct is composed of 5 β-strands: 3 N-terminally to the Δ114–139 deletion site (where the predicted hydrophobic helix would usually reside) and two forming a β-hairpin at the C terminus (Additional file 9: Figure S9a). Supporting these data, the residues assigned to the LITAF domain displayed greater ^1^H chemical shift distributions (~7.4 to 9.6 ppm), typical of β-sheet residues (Fig. 5), compared to the residues assigned to the N-terminal proline-rich region, which, in contrast, displayed a narrower ^1^H chemical shift pattern (between 7.8 and 8.4 ppm), suggesting the adoption of a random coil character.

The dynamic qualities of the LITAF Δ114–139 construct were next analysed by performing heteronuclear NOE (hetNOE) experiments. These experiments measure the motion of each assigned residue on a picosecond time scale. Positive hetNOE values close to the value of 1.0 represent slow picosecond motion, which is typically seen when protein domains adopt rigid secondary structure motifs, while small, or even negative, hetNOE values result from fast picosecond motion as seen in unstructured loops and at the flexible termini of proteins. The hetNOE analysis of the Δ114–139 construct, illustrated in (Additional file 9: Figure S9b), demonstrated that four of the five β-strands within the LITAF domain are rigid in the picosecond time scale, while in comparison, the first, most N-terminal β-strand, showed increased motion, and hence flexibility. In contrast, the proline-rich N-terminus showed mainly fast picosecond motion reminiscent of an unstructured protein domain. Interestingly, these analyses also revealed an area of reduced flexibility between residues 22 and 34, a region known to recruit interacting proteins to the LITAF molecule (Additional file 4: Figure S4a) [13].

### NMR-based structural model

**Fig. 6:**
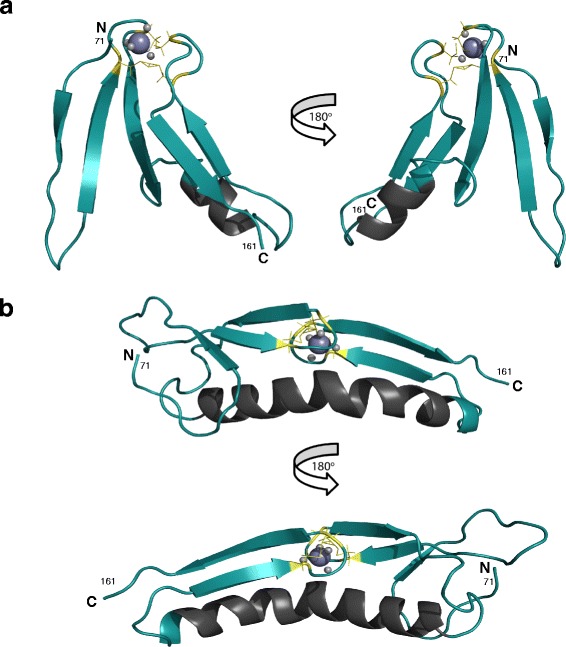
Rosetta structural models of LITAF. **a** Rosetta model of the C-terminal LITAF domain region of the LITAF Δ114–139 construct. The residue numbers correspond to the wild type human sequence. The zinc-coordinating cysteine residues are coloured *yellow* and the zinc atoms are illustrated by *purple balls*. **b** Rosetta model of the wild-type C-terminal LITAF domain. The zinc-coordinating cysteine residues are coloured *yellow* and the zinc atoms are illustrated by *purple balls*. The models calculated satisfy the known structural data: zinc coordination, NOE restraints and β-strand boundaries, with both models adopting a rubredoxin or zinc ribbon fold

In order to further characterise the structure of the LITAF domain, nuclear Overhauser effect spectroscopy (NOESY) spectral data were collected. These experiments probe the proximity of amino acids through space, thereby providing indicative data concerning the tertiary structure of the protein. We used Rosetta, the protein structure prediction suite of programs, to produce a structural model of the LITAF domain as only a limited number of NOESY cross-peaks were collected. Although Rosetta can be used in its most basic form to predict a protein structure based only on its primary sequence [30], more advanced Rosetta applications allow additional restraint information to be applied during the model calculations, making it possible to build a structural model that satisfies all our experimental data, including metal coordination, secondary structure boundaries, topology and NOE restraints [31]. After fragment selection for the model calculation, completed using Rosetta-CS and the NMR-defined secondary structure boundaries, Rosetta metalloprotein relax protocols were implemented as previously described [32], where the four conserved cysteine residues fixed for zinc coordination were C96, C99, C148 and C151 (full length residue numbering). A total of 5000 decoys for each of the two sequences, wild type and the Δ114–139 construct, were calculated and the quality of the models analysed by clustering using Calibur [33]. Figure 6 shows the structural models of the LITAF Δ114–139 and full-length LITAF domains, representing the largest clusters of similar structures.

The NMR-based model of the LITAF Δ114–139 construct (Fig. 6a), generated from the experimentally determined restraints, illustrate the five β-strands of the LITAF domain: three in an antiparallel orientation at the N-terminus and a β-hairpin at the C-terminus. The conserved cysteine residues, which reside within loops located between β-strands 2 and 3, and between β-strands 4 and 5, are shown to coordinate a zinc atom. Although the five β-strands do not form a continuous sheet, they are in close proximity and are part of an overall compact domain. The glycine/serine linker, that has replaced the predicted hydrophobic in-plane membrane anchor in the LITAF Δ114–139 construct, has been modelled as a short helix by Rosetta. The secondary structure elements are again maintained in the wild type model of the LITAF domain (Fig. 6b), with the predicted hydrophobic in-plane membrane anchor modelled as a single helix by Rosetta. As the coordination of the zinc atom has been maintained, this structure is not as compact as the LITAF Δ114–139 construct, and results in the β-strands lying parallel to the predicted membrane-anchoring helix. The critical coordination of the zinc atom by two pairs of cysteine residues, as shown in both models, is consistent with the rubredoxin, or ‘zinc β-ribbon’ family of zinc finger structural motifs [21]. Efforts to crystallize the LITAF constructs for further structural analysis were unsuccessful.

### Recruitment of proteins to the N-terminal proline-rich region directs LITAF to specific endosomal structures

**Fig. 7:**
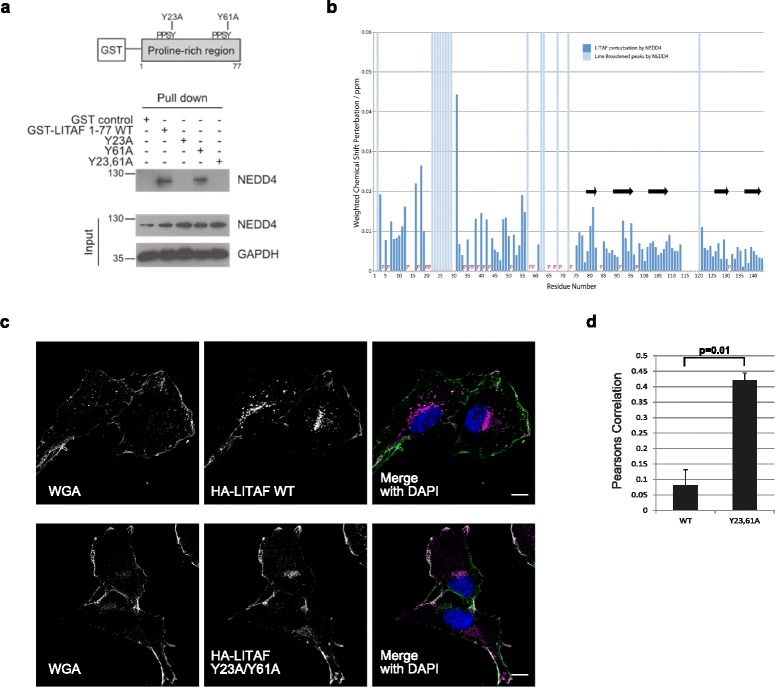
Recruitment of proteins to the proline-rich region determines the intracellular targeting of LITAF. **a** The first PPSY motif of LITAF (residues 20–23) interacts with the ubiquiting ligase, NEDD4. GST-LITAF 1–77 constructs (corresponding to the N-terminal proline-rich region), were recombinantly expressed and purified from *E. coli*, before incubation with cellular lysates prepared from retinal pigment epithelial (RPE) cells. A pull-down experiment was performed and the interacting proteins were separated by SDS-PAGE followed by western blotting. **b** The chemical shift perturbations seen in the LITAF Δ114–139 construct following incubation with recombinantly expressed NEDD4 596–944 (containing the 4 WW domains). A number of peaks are line-broadened indicating the interaction of the NEDD4 WW domains with these discrete regions of the LITAF proline-rich N-terminus. The interaction of NEDD4 with the proline-rich region had no effect on the C-terminal LITAF domain. **c** Representative images of confocal immunofluorescence microscopy of RPE cells stably expressing HA-LITAF wild type (WT) and HA-LITAF Y23A/Y61A. While HA-LITAF WT predominantly targets to endocytic vesicles, HA-LITAF Y23A/Y61A is mislocalised to the plasma membrane, labelled with wheat germ agglutinin (WGA). The merged images contain WGA coloured green and HA-LITAF coloured purple with areas of colocalisation appearing white. Scale bars denote 10 μm. **d** The colocalisation of HA-LITAF constructs with WGA was quantified using Volocity (Perkin Elmer) and a Pearson’s Correlation calculated; 10–13 images (each containing between four and five cells per field of vision) were taken from three biological replicates per cell line stably expressing the LITAF constructs. The Pearson’s correlation was calculated by the Volocity software with the threshold applied as previously described [87] (see Additional file 14 for individual data values). The graph shows the mean values with error bars denoting SEM. A *P* value of 0.01 was calculated using an unpaired two-tailed t-test. Significantly greater amounts of the HA-LITAF Y23,61A protein colocalised with WGA compared to wild type, confirming mislocalisation of the mutated construct towards the plasma membrane

Specific protein domains are known to interact with particular sequence motifs present in the LITAF N-terminal proline-rich region (Additional file 4: Figure S4a). Thus, the PPXY motif can bind the WW domains of the ubiquitin ligase, NEDD4 (Fig. 7a) [34], while the overlapping P(S/T)AP motif is predicted to interact with the UEV domain of the ESCRT-I component, TSG101 [35]. Our hetNOE data suggest that the N-terminal region of LITAF is mostly flexible, in contrast to the relatively rigid C-terminal membrane-anchoring LITAF domain. We next asked what effect the recruitment of proteins to the N-terminal proline-rich region has on the LITAF protein.

Based on the near complete (89%) assignment of the LITAF Δ114–139 construct, we probed the local interaction of LITAF with the WW domains of NEDD4. A comparison of the BEST-TROSY spectra of the ^15^N-labelled LITAF Δ114–139 in the presence and absence of recombinantly-expressed NEDD4 596–944 (containing the four WW domains) revealed the loss of a subset of amide cross peaks (Fig. 7b), most often caused by line broadening as a result of micromolar binding affinities. The missing signals correspond to residues 22–29 of the LITAF protein, which contain the PPSY motif known to interact with the WW domains of NEDD4, and are within a region of the N-terminal domain found to be less flexible in the hetNOE experiments. Of note, a second region between residues 57 and 72 of LITAF, which contains a second PPSY motif, showed peak broadening representing a potential additional NEDD4-WW domain interacting site. However, the functional significance of this second PPSY motif is uncertain, since it could not compensate for the loss of the first N-terminal PPSY motif in our pull-down experiments of endogenous NEDD4 from cellular lysates (Fig. 7a). Significantly, we did not detect any consequent structural changes to the C-terminal LITAF domain upon binding of NEDD4 to the N-terminal proline-rich region, showing that these regions of LITAF are structurally independent.

With no apparent structural effect on the C-terminal LITAF domain, we next asked whether the intracellular localisation of LITAF is influenced by the potential recruitment of proteins in vivo. HA-tagged LITAF constructs harbouring mutations that disrupt the PPSY motifs (Y23,61A) were expressed in retinal pigmented epithelial cells (where no endogenously expressed LITAF can be detected) and analysed by immunofluorescence microscopy (Fig. 7c). We found that disruption of the PPSY motifs led to significantly greater colocalisation of LITAF with the plasma membrane marker, wheat germ agglutinin, compared to the wild type protein (Fig. 7d). These data lead us to speculate that, while LITAF domains function as membrane anchors, the specific intracellular localisation is potentially influenced by the recruitment of interacting proteins to the relatively flexible and cytosol-facing N-terminal poly-proline arm.

### LITAF interacts specifically with PE head groups

**Fig. 8:**
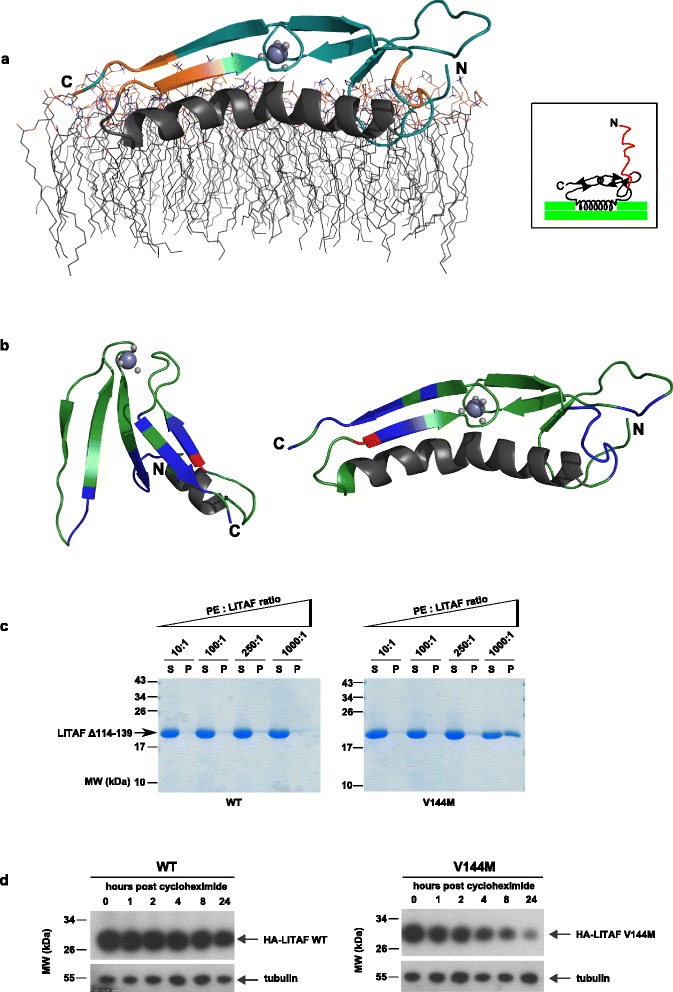
LITAF interacts with PE head groups and leads to protein instability in the V144M CMT1C-associated protein construct. **a** The position of residues displaying chemical shift perturbations greater than 1 standard deviation of the mean observed in the presence of PE head groups are coloured orange on the structural model of the LITAF domain. The illustration also shows the possible orientation of the LITAF domain as it inserts into a membrane containing phosphotidylethanolamine and phosphatidylcholine [85]. A cartoon diagram of the topology of the LITAF protein embedded in a lipid membrane bilayer (*green*), additionally showing the position of the flexible N-terminal proline-rich region (*red*), is shown in the inset. **b** The location of the residues displaying chemical shift perturbations greater than 1 standard deviation from the mean following the introduction of the V144M mutation are shown in blue on the structural models of the LITAF domain (LITAF Δ114–139 on the left and LITAF WT on the right). The position of Valine 144 is shown in red. **c** Equal amounts of LITAF Δ114–139 were incubated with increasing concentrations of PE for 2 hours at room temperature before centrifugation at 20,000 × *g* for 10 minutes to obtain soluble (S) and insoluble (P) fractions, respectively. The fractions were then separated by SDS-PAGE prior to staining with Coomassie Blue. In contrast to the WT construct (*left panel*), the V144M mutation rendered the protein unstable in the presence of higher concentrations of PE, leading to precipitation (*right panel*). **d** Retinal pigment epithelial cells stably expressing either HA-LITAF WT or HA-LITAF V144M were incubated with 300 μg/mL cycloheximide and incubated at 37 °C for the times indicated. Cellular lysates were prepared and the amount of HA-LITAF protein was determined by western blotting

As a zinc-binding structure anchored to the surface of membranes, distinct residues within the LITAF domain would be predicted to be in close proximity to phospholipid head groups. We therefore asked whether particular residues contained within the LITAF domain are able to interact with phospholipid head groups in solution. To this end, ^15^N-labelled LITAF Δ114–139 was incubated with phosphoglycerol (PG), phosphocholine (PC), PE, phosphoserine (PS) and the polyphosphatidylinositol head group mimic, inositolhexakisphosphate (IP_6_) [36–39], and analysed by NMR in molar ratios up to 1:1000 protein:head group (Additional file 10: Figure S10). While no interaction was seen between LITAF and PG, PC, PS or IP_6_, we identified discrete interactions between specific residues located in the C-terminal LITAF domain and PE head groups. As expected from the structural model, these PE-interacting residues reside within regions of the LITAF domain that would be predicted to be in close proximity with the surface of membranes (Fig. 8a and Additional file 11: Figure S11a), lending further support to the validity of our model.

### The V144M CMT1C-associated mutation leads to subtle changes in the LITAF Δ114–139 construct and disrupts the interaction with PE

All known CMT1C-associated pathogenic mutations in LITAF are found in the C-terminal LITAF domain. Most mutations reside within the predicted hydrophobic anchor region and are predicted to disrupt the helical propensity of this specific part of the protein (Fig. 1 and Additional file 12: Figure S12a) [40–42]. However, as stated above, experimental study by NMR spectroscopy of constructs containing the predicted hydrophobic helical region were not possible. Nevertheless, the LITAF Δ114–139 construct used to generate the structural model does contain valine at position 144, a residue that, when mutated to a methionine (V144M), is known to cause CMT1C [43]. The V144M mutation does not affect membrane association and its punctate staining pattern by immunofluorescence microscopy is consistent with retained endosomal targeting (Additional file 12: Figure S12b, c). We therefore set out to characterise the effects of this pathogenic mutation by solution NMR spectroscopy. Additional file 11: Figure S11b shows the chemical shift perturbations that are manifested by the V144M mutation in the Δ114–139 construct when compared to the wild type counterpart. If the amino acid substitution had little effect on the construct, we would expect that only signals corresponding to residues in close proximity to the site of mutation would be perturbed. However, dispersed chemical shift perturbations were seen as a result of this CMT1C mutation, including within the region of the N-terminal β-sheets on the opposite side of the deleted hydrophobic anchor. These data therefore suggest that the V144M pathogenic mutation causes subtle biochemical and biophysical changes whilst maintaining the overall secondary structure of the LITAF domain, pointing towards an additional factor that must lead to clinical disease.

By analysing the residues that show the greatest chemical shift perturbations following the introduction of the V144M mutation, we noted that these residues correspond to those that interact with PE head groups (Fig. 8a, b and Additional file 11: Figure S11b). We therefore asked whether the interaction of the LITAF domain with PE is altered by the introduction of the V144M mutation. Indeed, we found that LITAF Δ114–139 V144M interacts with PE at lower head group concentrations (1:250 molar ratio) than the wild type construct whilst utilising the same specific residues (Additional file 13: Figure S13). However, while the V144M construct – similarly to the LITAF Δ114–139 WT protein – remained soluble and un-degraded for periods of many months when stored in isolation, this mutant construct became rapidly insoluble when incubated with PE at 1:1000 molar ratio, leading to the disappearance of the assigned peaks from the NMR spectra and visible precipitation (Fig. 8c and Additional file 13: Figure S13). In contrast, wild type LITAF Δ114–139 remained consistently soluble and folded at these concentrations of PE. To determine the effect of the V144M mutation on the stability of LITAF in vivo, we performed cycloheximide chase experiments in retinal pigment epithelial (RPE) cells stably expressing either wild type HA-LITAF or HA-LITAF V144M. In keeping with our in vitro findings, HA-LITAF harbouring the V144M mutation was degraded more rapidly compared to the wild type construct (Fig. 8d). These data therefore suggest that the V144M pathogenic mutation alters the interaction between the LITAF domain and PE head groups, pointing towards an acquired misfolded state on the surface of membranes, and potentially deleterious effects on intracellular membranes involved in endocytic traffic.

## Discussion

Despite the extensive architectural conservation of LITAF domains across eukaryotes, our report is the first to characterise this ancient domain structurally. In contrast to the assertion made by Lee et al. [11] that LITAF domains are C-terminally inserted into membranes, our data show that the hydrophobic region does not traverse membranes, demonstrating that both the N-terminal and C-terminal halves of the LITAF domain reside on the cytosolic surface of membranes, in agreement with an earlier proposal made by Ponting et al. [15] in 2002 and the recent publication by Qin et al. [44]. The LITAF domain consists of five β-sheets, three N-terminal and two C-terminal to the predicted hydrophobic anchor region, and is stabilised by the coordination of a zinc atom by two pairs of evolutionarily-conserved cysteine residues. In contrast, the N-terminal proline-rich region is mostly unstructured and behaves independently of the C-terminal LITAF domain. We show that, far from being an inert component of the full length protein, the ability of the proline-rich region to bind to other cytosolic proteins appears to have a significant influence on intracellular targeting. These data therefore support the notion that, while LITAF domains serve as intracellular membrane anchors, the precise function and localisation is determined by the proteins that interact with the proline-rich N-terminal arm. This last point might help explain the many contradictory data that currently exist concerning the specific intracellular localisation of LITAF-domain-containing proteins in a variety of cell types [9–11, 13, 18].

Consistent with a protein domain that resides in close proximity to membranes, we also show that specific residues within the LITAF domain interact with PE head groups. PE is the second most abundant phospholipid (behind PC) in mammalian cells, accounting for, on average, approximately 25% of cellular phospholipids. PE is found across all intracellular membranes and, as a consequence of its cone-shape, has been proposed to play an essential role in membrane fusion and curvature [45]. By accommodating and interacting with PE head groups, it is tempting to speculate that LITAF domains might therefore play further roles in the regulation of membrane invagination and tubulation, which are essential for the sorting and recycling of membranous cargoes on endosomes [46].

Our data are consistent with LITAF being a monotopic membrane protein anchored to membranes via an in-plane helical membrane anchor, present within the LITAF domain [27]. However, efforts to analyse the full length LITAF protein, or the predicted hydrophobic helical anchor region in isolation, either by solution NMR or crystallography, were unsuccessful, likely due to the inability of the tested detergent and lipid micelles to mimic the membrane environment found intracellularly. Furthermore, the molecular weight of LITAF remains too small for current cryo-EM techniques [47]. This again highlights the general issue in structural biology that, despite the fact that membrane proteins make up approximately 30% of all eukaryotic cellular proteins [48] and constitute around 60% of current drug targets [49], membrane proteins still account for less than 1% of structures in the Protein Data Bank [50]. Moreover, none of these current membrane protein structures contained within the database demonstrate in-plane membrane anchoring domains as we propose for LITAF. In the absence of a predicted helix-loop-helix ‘hairpin’ motif that inserts into the lipid bilayer as postulated for other integral membrane proteins [51], we can therefore only speculate at present on how precisely the predicted hydrophobic helical anchor associates with membranes. However, our data regarding the loss of membrane association following the substitution of eight hydrophobic amino acids within the predicted helix, in conjunction with our structural data, lead us to speculate that this anchoring-region embeds into the cytosolic-facing monolayer of the membrane bilayer by adopting an amphipathic character [52, 53].

Schwann cells are predicted to be the principal site of pathology in the demyelinating subtypes of CMT. Due to the significant number of genes associated with demyelinating CMT that are predicted or known to encode proteins that function on endocytic pathways [5], the critical importance of intracellular membrane trafficking in maintaining peripheral nerve myelination by Schwann cells has been brought to the fore. Most mutations in these demyelinating CMT genes associated with membrane trafficking cause disease in an autosomal recessive manner, pointing towards loss of cellular function as the underlying pathogenic mechanism in these instances. In contrast, CMT1C associated with mutations in *LITAF* is inherited in an autosomal dominant pattern. Previous reports have confirmed that the expression of LITAF is not critical for peripheral nerve myelination in mice [54], making the ‘dominant-negative’ hypothesis, due to the possible formation of non-functional LITAF hetero-oligomers (containing wild type and mutant species), less likely. On the contrary, a more probable pathogenic mechanism involves a ‘toxic gain-of-function’ resulting from the altered molecular and cellular properties of mutant LITAF when expressed in vivo [55]. Significantly, the LITAF protein is known to be widely expressed across tissues [11], suggesting that, while compensatory mechanisms must exist in most cell types, the intracellular membrane trafficking pathways in Schwann cells must be distinctly susceptible to the presence of a mutant copy of LITAF.

But how do CMT1C point mutations cause toxicity? Thus far, all proven CMT1C-associated pathogenic mutations in LITAF are found in the C-terminal LITAF domain and previous reports have suggested that these mutations might disrupt the association of LITAF with membranes [11], although the precise mechanism remained unclear. Here, we show that the CMT1C-associated pathogenic V144M mutation in LITAF leads to chemical shift perturbations across the LITAF domain that sits in close approximation to the surface of membranes, whilst maintaining an overall secondary structure similar to the wild type protein. However, when the V144M construct was incubated with PE head groups, the disease-associated protein became misfolded, leading to precipitation in vitro, in complete contrast to wild type LITAF. Furthermore, cycloheximide chase experiments in vivo suggested that the V144M construct was degraded more rapidly compared to wild type. It is therefore tempting to speculate that all CMT1C mutations might lead to similar structural alterations within the LITAF domain, resulting in an unstable and toxic interaction with lipid head groups and consequent endocytic membrane disruption [10, 56]. This aberrant association of LITAF with membranes might lead to the dysfunctional trafficking of Schwann cell membrane receptors, including growth factor receptors [9, 57, 58] and cell adhesion molecules [6, 59], with consequent disruption of the critical endocytic pathways that maintain the integrity of peripheral nerve myelin. Such mistrafficking would, in turn, lead to instability of the myelin sheath, demyelination, secondary axonal degeneration and, eventually, the motor and sensory deficits that characterise patients affected by a diagnosis of CMT1C.

## Conclusions

In summary, we present the first structural protein model of LITAF based on experimental data, and describe the biophysical and biochemical consequences of a CMT1C-associated pathogenic mutation. We anticipate that this work will form a platform for the future study of LITAF, and LITAF-domains in general, and their role in intracellular membrane trafficking. In the absence of any current treatments for CMT, we believe that such investigation will not only lead to the identification of future therapeutic strategies for CMT1C, but will also highlight molecular and cellular dysfunctional pathways common to more than one subtype of this common, currently incurable, neurological disorder.

## Methods

### Reagents and cell culture

Antibodies and reagents used during this study include mouse anti-LITAF (BD Biosciences, #611614, RRID: AB_399056, Lot #29025), rabbit anti-VPS26 (gift from M. N. J. Seaman, Cambridge UK), mouse anti-LAMP1 (DSHB, H4A3, RRID: AB_2296838), mouse anti-EEA1 (BD Biosciences, #610457, RRID: AB_397830, Lot #5023919), rabbit anti-TGN46 (Abcam, ab50595, RRID: AB_2203289, Lot #GR133666-6), mouse anti-HA (16B12, Covance, mms-101R-50), anti-myc (9B11, Cell Signaling, #2276S, RRID: AB_10693333, Lot #19), rabbit anti-Flag (Cell Signaling, #2368, RRID: AB_2217020, Lot #6), rabbit anti-calnexin (Santa Cruz, sc-11397, RRID: AB_2243890, Lot #F1808), rabbit anti-NEDD4 (Abcam, ab14592, RRID: AB_301364), mouse anti-GAPDH (Sigma, G8795, RRID: AB_1078991, Lot #092M4820V), mouse anti-β-lactamase (Abcam, ab12251, RRID: AB_298974), mouse anti-α-tubulin (DSHB, AA4.3, RRID: AB_579793, Lot #8/29/13), rabbit anti-GFP (Thermo Fisher Scientific, A11122, RRID: AB_10073917, Lot #1453341), CF488A Wheat Germ Agglutinin (Biotium, #29022-1, Lot #15 W0225), Alexa Fluor 488- (Thermo Fisher Scientific, A11034, RRID: AB_2576217, Lot #939304), Alexa Fluor 568-conjugated goat anti-rabbit H + L (Thermo Fisher Scientific, A11036, RRID: AB_10563566, Lot # 757102), goat anti-mouse H + L (Thermo Fisher Scientific, A11031, RRID: AB_144696, Lot #1126619), Alexa Fluor 488-conjugated goat anti mouse IgG_1_ (Thermo Fisher Scientific, A21121, RRID: AB_2535764, Lot #1485220), and Alexa-Fluor 555-conjugated anti-mouse IgG_2a_ (Thermo Fisher Scientific, A21137, RRID: AB_2535776, Lot # 1541489). HRP-conjugated goat anti-rabbit and goat anti-mouse antibodies were used as secondary antibodies for western blotting (Sigma-Aldrich). Proteins separated by SDS-PAGE followed by transfer to nitrocellulose membranes were detected using WesternBright ECL western blotting detection kit (Advansta, CA, USA).

Full length cDNAs for LITAF (IMAGE clone 4000250) and NEDD4 (IMAGE clone 8860509) were obtained from Source Bioscience (Cambridge, UK). LITAF Δ114–139 and LITAF N-helix constructs were generated using synthesized gBlocks (Integrated DNA Technologies, Iowa, USA) and Gibson cloning into the relevant expression vectors (NEB) [60]. Site-directed mutagenesis was performed using a QuikChange II XL kit (Stratagene). All DNA constructs were sequenced and validated by Source Bioscience (Cambridge, UK).

HeLa cells (gift from F. Buss, Cambridge UK) were grown at 37 °C in RPMI (Sigma-Aldrich) containing 10% FCS and 2 mM L-glutamine, 100 U/mL penicillin and 100 μg/mL streptomycin in a 5% CO_2_ humidified atmosphere. RPE cells (ARPE-19, ATCC) were grown at 37 °C in DMEM:Ham’s F-12 (50:50) (Sigma-Aldrich) containing 10% FCS and 2 mM L-glutamine, 100 U/mL penicillin and 100 μg/mL streptomycin in a 5% CO_2_ humidified atmosphere. Cells were transfected using polyethylenimine (Polysciences, PA, USA) or Lipofectamine LTX (Thermo Fisher Scientific).

### Comparative genomics and phylogenetics

Characterised LITAF domain protein sequences from *H. sapiens* were used as initial queries to identify putative LITAF domain proteins from the genomes of the organisms listed in (Additional file 1: Figure S1). Where no homologue was identified, secondary searches were carried out using identified sequences from more closely related taxa. Genome searches were carried out using the basic local alignment search tool (BLAST) against publicly available genome databases accessible via NCBI [61] with exception of those listed below; *Plasmodium falciparum* (PlasmoDB, [62]); *Toxoplasma gondii* (ToxoDB, [63]); *Naegleria gruberi*, *Guillardia theta*, *Emiliania huxleyi*, *Bigelowiella natans*, *Phaeodactylum tricornutum, Phytophthora sojae, Psuedo nitzschia multiseries*, *Populus trichocarpa*, *Selaginella moellendorffii*, and *Chlamydomonas reinhardtii* (Joint Genome Initiative, US Department of Energy [64]); *Bodo saltans* (GeneDB [65]); *Leishmania major*, *Trypanosoma cruzi*, and *Trypanosoma brucei* (TriTrypDB [66]); and *Giardia lamblia* (GiardiaDB [67]). All identified LITAF domain sequences were verified by reciprocal BLAST against the non-redundant protein database (NCBI). For phylogenetic reconstruction, protein sequences were aligned using MAFFT via jalview [68, 69] and edited manually to remove gaps and poorly conserved regions. Phylogenies were reconstructed using Bayesian (MrBayes) and bootstrapped maximum likelihood (RaxML, PhyML) approaches. PhyML was run through the South of France Bioinformatics Platform web server [70]. RaxML and MrBayes were run through the Cyberinfrastructure for Phylogenetic Research (CIPRES) Science Gateway web server [71]. MrBayes version 3.2.2 analyses were run using a mixed model for 1 × 10^6^ generations, with convergence verified by standard deviation of split frequencies < 0.05. All trees before plateau were removed as burn-in. The appropriate evolutionary model was assigned using protest v2.4 [72]. Domain prediction was carried out via the NCBI conserved domain database (CDD), HMMScan (HMMER, Janelia) [73] and InterProScan [74].

### Immunofluorescence microscopy

For general immunofluorescence microscopy, cells were plated on glass coverslips, fixed in 4% paraformaldehyde and permeabilised with 0.1% TritonX-100 in phosphate buffered saline (PBS) at pH 7.4. Bovine serum albumin (BSA; 1%) in PBS was used to block fixed cells before incubation with primary antibodies followed by AlexaFluor-conjugated secondary antibodies. Cover slips were mounted onto glass slides using ProLong Gold Antifade Mountant with DAPI (Thermo Fisher Scientific). Wide-field fluorescent images were obtained using a Zeiss Axioimager Z2 Upright Wide-field microscope (Carl Zeiss), and confocal images were acquired using Zeiss LSM710 and LSM880 confocal microscopes (Carl Zeiss). Imaging data were analysed using Zeiss ZEN software (Carl Zeiss) and Volocity (PerkinElmer).

### Membrane fractionation and TX114 extraction

To isolate cellular membranes, HeLa cells were washed with cold 10 mM Tris pH 7.4 before scraping into a small volume of 10 mM Tris pH 7.4, 10 mM NaCl and 1.5 mM MgCl_2_. The cells were then lysed by gently passing through a 25G needle multiple times before centrifugation at 1330 *g* for 10 minutes. The resulting supernatant was then sonicated briefly on ice before further centrifugation at 280,000 *g* for 45 minutes to obtain a membrane fraction (pellet) and cytosolic supernatant. The membranes were resuspended in an equal volume of buffer to the supernatant before proceeding to SDS-PAGE and western blotting. For the LITAF cysteine mutants, the membrane pellets were additionally incubated with 10 mM Tris pH 7.4, 10 mM NaCl, 1.5 mM MgCl_2_ and 1% TX100, and complete protease inhibitor (Roche) to extract soluble membrane proteins before a second centrifugation at 280,000 *g* for 45 minutes. The soluble supernatant was transferred to a new tube and the insoluble pellet was resuspended with an equal volume of 10 mM Tris pH 7.4, 10 mM NaCl and 1.5 mM MgCl_2_ containing protease inhibitors before proceeding to SDS-PAGE and western blotting. Phase partitioning of endogenous LITAF and marker protein from HeLa cell membranes with Triton X114 (Sigma-Aldrich) was performed as previously described [75], with equal proportions of the detergent and aqueous phases being analysed by western blotting.

### Determination of LITAF membrane topology

HeLa cells stably expressing HA-LITAF-myc were generated using the pLXIN retroviral system (Clontech) as previously described [76], and a single transduced clone selected for further study to ensure consistency across experiments. Cells were seeded onto glass cover slips and left to settle overnight. The following steps until fixation with paraformaldehyde were performed on ice at 4 °C. The cells were first washed twice with ice-cold KHM buffer (20 mM HEPES pH 7.4, 110 mM potassium acetate, 2 mM MgCl_2_). Digitonin (20 μM; Sigma-Aldrich) was then used to permeabilise the plasma membrane by incubating for 3 minutes on ice before washing three times with ice-cold KHM buffer. Free aldehydes where then quenched with 50 mM NH_4_Cl in PBS for 5 minutes before twice washing with PBS. Cells were next blocked with 1% BSA in PBS for 30 minutes before incubation with primary antibodies for 1 hour on ice at 4 °C. Following three further washes with PBS, the cells were fixed with 4% paraformaldehyde at room temperature for 15 minutes, washed with PBS and incubated with AlexaFluor-conjugated secondary antibodies before mounting on glass slides for immunofluorescence microscopy. Digitonin (100 μM) was used to permeabilise both endosomal membranes and plasma membranes, additionally exposing epitopes on the luminal side of endocytic vesicles.

For cell-free expression of HA-LITAF-myc, a rabbit reticulocyte lysate cell-free expression system was used in the presence of canine microsomal membranes (Promega). The signal peptide-containing protein, β-lactamase, served as a control for membrane insertion and translocation into the lumen of microsomes, and hence protection from added protease digestion. The expressed proteins were incubated with proteinase K (NEB) for 1 hour at 37 °C before separation by SDS-PAGE followed by western blotting.

### Bacterial protein expression and purification

cDNAs corresponding to wild type LITAF, Δ114–139 and N-helix were cloned into the pOPINS vector before expression of the resulting His_6_-SUMO-tagged protein constructs in C43(DE3) BL21 *E. coli*. Bacterial lysates were prepared by cell disruption of the bacterial pellet in lysis buffer (50 mM HEPES pH 7.4, 300 mM NaCl, 3 mM 2-mercaptoethanol, 200 μM AEBSF) followed by centrifugation of insoluble material at 100,000 *g* for 30 minutes. His_6_-SUMO-LITAF constructs were purified from lysates using Ni-NTA resin (Qiagen) and the eluting imidazole removed using Centripure P25 Zetadex gel filtration columns (Generon) and the protein buffer-exchanged into 20 mM HEPES pH 7.4, 100 mM NaCl, 0.5 mM TCEP. The purified protein was then incubated with ULP1 SUMO protease for 1 hour at room temperature and the His_6_-SUMO tag removed by a second incubation with Ni^2+^-NTA. The final purification step consisted of size exclusion chromatography using a Superdex 200 10/300 gel filtration column (GE Healthcare Life Sciences). Protein samples were concentrated using Vivaspin centrifugal concentrators (Sartorius, UK) and any insoluble particles removed using Proteus clarification mini spin columns (Generon). For NMR analysis, proteins were expressed in C43(DE3) BL21 *E. coli* grown in minimal media containing ammonium-^15^N chloride (Sigma-Aldrich) and/or ^13^C-D-Glucose (Cambridge Isotopes) as previously described [77]. For wild type LITAF constructs (containing the predicted hydrophobic helical membrane anchor), detergents were required at each step to maintain solubility. For the experiments described in this study, 1% DDM (w/v) or 1% FC12 were used for cell lysis and 0.04% DDM (w/v) or 0.125% FC12 added to each subsequent buffer when dealing with the full length wild type LITAF protein. For GST-tagged proteins, cDNA encoding residues 1–77 of human LITAF and residues 596–944 of human NEDD4 were cloned into pGEX-4 T1 expression vector (GE Healthcare Life Sciences) for expression in C43(DE3) BL21 *E. coli*. Purification was performed using glutathione sepharose 4B according to the manufacturer’s instructions (GE Healthcare Life Sciences). For NMR analysis, purified GST-NEDD4 596–944 was buffer-exchanged into 20 mM HEPES pH 7.4, 100 mM NaCl and 0.5 mM TCEP by using a Superdex 75 10/300 gel filtration column (GE Healthcare Life Sciences).

### GST pull-down assays

Cell lysates were prepared by scraping RPE cells into a small volume of lysis buffer (20 mM HEPES pH 7.4; 150 mM NaCl; 1 mM EDTA; 0.5% Triton X-100 (v/v); complete protease inhibitor (Roche)) and transferring into a 1.5 mL microcentrifuge tube. The lysate was then sheared by gently passing the solution back and forth through a 21-G needle followed by a 25-G needle, before centrifugation at 20,000 *g* for 30 min at 4 °C. The resulting supernatant was first precleared by gentle agitation for an hour at 4 °C with glutathione sepharose 4B beads (GE Healthcare Life Sciences) before the addition of GST-LITAF 1–77 constructs at a concentration of 0.2 mg/mL. Following a further incubation for 2 hours at 4 °C with gentle agitation, glutathione sepharose 4B beads were added and incubated for 1 hour more with gentle agitation at 4 °C. The beads were washed twice with lysis buffer and once with PBS before elution of the bound proteins by heating in SDS sample buffer (6 M Urea, 1% SDS (w/v), 1 M 2-mercaptoethanol and 150 mM Tris (pH 6.7)). Eluted proteins were separated by SDS-PAGE and transferred to nitrocellulose membranes for western blotting.

### Cycloheximide chase

RPE cells stably expressing wild type HA-LITAF or HA-LITAF V144M were seeded before incubation with 300 μg/mL of cycloheximide (Sigma). Cells were lysed in RIPA buffer at 0, 1, 2, 4, 8 and 24 hours after incubation with cycloheximide before brief sonication and centrifugation at 20,000 *g* for 30 minutes at 4 °C. The supernatants were transferred to new tubes and the protein concentration determined using Precision Red reagent (Cytoskeleton, Inc.). Equal amounts of protein were separated by SDS-PAGE before analysis by western blotting.

### MicroPIXE

The unambiguous identity of the metals in the recombinantly expressed LITAF proteins and their stoichiometry was determined using microPIXE (microbeam Proton Induced X-ray Emission), an established technique for such analyses [78]. The recombinant proteins were prepared as described above before buffer exchange into 10 mM Tris pH 7.4, 200 mM NaBr and 0.5 mM TCEP using a Superdex 75 GL size exclusion chromatography column (GE Healthcare Life Sciences). DDM (0.04%) was also used to maintain the solubility of wild type LITAF constructs for the purpose of these experiments. Resulting protein concentrations, estimated by measuring the absorbance at 280 nm, were 1.7 mg/mL, 4.7 mg/mL and 1.7 mg/mL for wild type LITAF, LITAF Δ114–139 and LITAF N-helix, respectively. This pre-measurement buffer exchange was also necessary to remove any traces of sulfur and chlorine. For micro-PIXE measurements of proteins, sulfur acts as an internal standard, and because of the proximity of the X-ray emission energies of sulfur and chlorine to one another, strong chloride fluorescence can affect the accuracy with which the sulfur peak can be quantified [78]. The measurements were carried out at the Ion Beam Centre, University of Surrey, UK [79]. A 2.5-MeV proton beam of 3 μm in diameter was used to induce characteristic X-ray emission from dried liquid protein droplets (volume per droplet = 0.1 μL) under vacuum. The X-rays were detected in a solid state lithium drifted silicon detector with high energy resolution. By scanning the proton beam in *x* and *y* over the droplet, spatial maps were obtained of all elements heavier than magnesium present in the sample. Quantitative information was obtained by collecting 3- or 4-point spectra from each droplet. These spectra were analyzed with GUPIX [80] within DAN32 [81] to extract the relative amount of each element of interest in the sample.

### NMR experiments and analysis

^15^N- and ^13^C-labelled proteins were prepared in 20 mM HEPES pH 7.4, 100 mM NaCl, 0.5 mM Tris(2-carboxyethyl)phosphine hydrochloride (TCEP) at typical concentrations of between 60 and 250 μM. All experiments were collected at 298 K on Bruker Avance III 600 MHz and Avance 2+ 700 MHz spectrometers equipped with cryogenic triple resonance TCI probes. Data were processed using the software packages Topspin 3.2 (Bruker) and both Multi-Dimensional Decomposition [82] and Compressed Sensing [83] for data collected by Non Uniform Sampling. All NMR data were analyzed using SPARKY 3 (T. D. Goddard and D. G. Kneller, University of California, San Francisco).

1D proton transverse relaxation (T2) experiments were used to estimate the molecular weight of the LITAF domain. For a globular protein, its transverse relaxation is governed by its molecular weight by the following relationship: MW × 0.5 ≈ 1/5 × T2, where T2 is in seconds and MW is in kDa. Standard 1D proton transverse relaxation experiments were collected with different delays (1 and 2), with observed peak intensities reduced after an increased delay. T2 can therefore be estimated using the following equation: T2 = 2(Δ_1_–Δ_2_)/ln(I_1_/I_2_), where I is the peak intensity and Δ is the relaxation delay.

Backbone chemical shift assignments were completed using HNCO, HNCACO, HNCA, HNCACB and CBCACONH. The assignment of proline residues was aided by ^13^C observed CON experiments complimented with HCBCANCO, HCBCACON, HNCANNH and HNCOCANNH experiments. Side chain HA and HB were assigned using an HBHACONH experiment. Through space contacts were established by ^15^N and ^13^C HSQC NOESY experiments.

Secondary structure predictions were made using N, HN, CA, CB and HA chemical shifts and TALOS+ [29].

Assigned BEST-TROSY (band selective excitation short transients transverse relaxation optimized spectroscopy) were used to make weighted chemical shift maps on mutation and head group or NEDD4 WW domain interactions using the equation ((Δ^1^H)^2^ + (Δ^15^N/5)^2^)^0.5^, where the Δ denotes the difference in parts per million of the chemical shift between the assigned apo peak and its nearest neighbour in the mixed sample spectra.

In the head group experiments, PG, PC, PS, PE and IP_6_ head groups were sourced from Sigma-Aldrich and incubated with LITAF (60 μM concentration) at molar ratios of 1:1000 (PG, PC, PE) and 1:500 (PS, IP_6_). The pH of each LITAF/head group mixture was checked and found to be the same as the sample used to generate the control spectra.

### LITAF structure modelling

NMR assignment information was submitted to the CS Rosetta webserver for *de novo* structure calculations [31, 84], which did not return a convergence of structures, suggesting a low confidence in the prediction. Given the established metal coordination of the LITAF constructs and NMR restraints, a zinc-bound structure model methodology was then followed using CS-Rosetta fragment files with the abinitio.metalrelax protocol of the Rosetta package [32]. The jump restraint file was edited so that the zinc atom coordinated cysteine residues C96, C99, C148 and C151 (full length residue numbers). Running the scripts for both the LITAF Δ114–139 and wild type LITAF domains returned 5000 structural models for each construct (also referred to as decoys) of improved confidence. These decoys were clustered with a 5 Å cutoff using Calibur [33] and scored for similarity to the centre of each cluster using the score_jd2 script that is part of the Rosetta package. The structures shown in Figs. 6 and 8 were representative of the lowest energy structures of the largest cluster in each calculation round.

## Additional files

Additional file 1: Figure S1. LITAF domains are conserved across the eukaryotes. Coulson plot showing the distribution of LITAF domain-containing proteins across the eukaryotes. Filled circles indicate the presence of a gene encoding a protein with an identifiable LITAF domain. Unfilled circles indicate taxa where no LITAF domain proteins could be identified. Numbers within circles indicate the number of distinct genes encoding proteins bearing LITAF domains. (PDF 77 kb)
Additional file 2: Figure S2. Phylogenetic reconstruction of the evolution of LITAF domain proteins within the chordates. The best Bayesian topology is shown (MrBayes) with branch support for key nodes indicated according to the key. (PDF 33 kb)
Additional file 3: Figure S3. Predicted domain organisation of representative LITAF domain proteins from across the eukaryotes. (PDF 36 kb)
Additional file 4: Figure S4. Sequence of human LITAF and alignment of representative eukaryote LITAF domains. (a) Amino acid sequence of human LITAF. Residues comprising the C-terminal LITAF domain are outlined in red. The two PPSY motifs that are predicted to interact with WW domains, and the PSAP motif that can associate with the UEV domain of TSG101 that are present in the N-terminal proline-rich region are outlined by blue and green boxes, respectively. (b) LITAF domain alignment constructed from representative taxa encompassing the diversity of eukaryotes. Residues are numbered according to the human LITAF sequence and coloured according to the Clustalx scheme as implemented in the Jalview program to highlight conserved amino acid properties. Shading indicates the degree of conservation. The conserved cysteine residues are coloured red and denoted with asterisks. (c) Helical wheel alignment of residues 113–134 of LITAF, predicted to form a helix by Jpred4 [86], to illustrate the predicted clustering of hydrophobic residues on one face of the helix, compatible with an amphipathic helix. (d) Helical wheel alignment of residues 113–134 of LITAF, with the hydrophobic residues on one face of the helix substituted with asparagine (N), as found in the soluble LITAF N-helix construct. (PDF 1131 kb)
Additional file 5: Figure S5. Intracellular localisation of LITAF. Colocalisation immunofluorescence studies of wild type (WT) LITAF constructs stably expressed in retinal epithelial cells (RPE) by confocal microscopy. Epitope-tagged LITAF WT targeted to punctate structures with partial colocalisation with markers of early endosomes (EEA1, Rab5), late endosomes/lysosomes (CD63, LAMP1) and with the retromer component, VPS26. Minimal colocalisation of HA-LITAF WT with the trans Golgi network marker (TGN46) or plasma membrane marker, wheat germ agglutinin, was observed. Scale bar denotes 10 μm. (PDF 3672 kb)
Additional file 6: Figure S6. (a) Schematic diagram illustrating the position of the conserved cysteine residues mutated to alanine in the N-term AxxA and C-term AxxA HA-LITAF constructs. (b) HA-LITAF constructs were transiently expressed in HeLa cells and visualised by immunofluorescence confocal microscopy. In contrast to the wild type LITAF, which targeted multiple punctate structures consistent with endosomes (HA-LITAF WT, left panel), the LITAF constructs in which the N-terminal or C-terminal conserved cysteine pairs were mutated to alanine (HA-LITAF N-term AxxA and HA-LITAF C-term AxxA) appeared in large puncta, consistent with them being proteins prone to aggregation. Nuclei were stained with DAPI. Scale bars denote 10 μm. (c) Membrane fractionation from HeLa cells transiently expressing HA-LITAF constructs. A soluble fraction (Soluble) was obtained following high speed centrifugation. The resulting membrane pellet was further incubated with 1% TX100 in order to extract soluble membrane proteins (TX100) from insoluble material (Pellet). Calnexin and tubulin were used for control purposes as examples of integral membrane and soluble proteins, respectively. While HA-LITAF WT was found in the TX100 solubilised membrane fraction similarly to calnexin, HA-LITAF construct harbouring mutations in either the N-terminal or C-terminal conserved cysteine pairs within the LITAF domain were found predominantly in the TX100 insoluble pellet (P), consistent with protein aggregation. Note that longer exposure times were required for the cysteine mutant samples as the expression level of these mutated proteins was consistently lower compared to wild type. (PDF 246 kb)
Additional file 7: Figure S7. (a) Representative traces of recombinantly-expressed LITAF wild type (WT; green), LITAF N-helix (red) and LITAF Δ114–139 (blue) subjected to size exclusion chromatography (SEC) using a Superdex 200 10/300 GL column (GE Healthcare Life Sciences). The broad peak on the SEC column corresponding to LITAF WT indicated the presence of higher order oligomers. (b) Fractions from the size exclusion chromatography of LITAF WT (arrows labelled 1–10 in (a)) were separated by SDS-PAGE and visualised with InstantBlue (Expedeon). A protein band corresponding to LITAF WT was present in fractions 2–10. (c) Western blot of fractions 2–10 from the size exclusion chromatography of LITAF WT separated by native gel electrophoresis before transfer to a nitrocellulose membrane and immunoblotted for LITAF. Note that LITAF WT exists as larger molecular weight species in the earlier fractions compared to the later fractions, consistent with the size exclusion chromatography trace seen in (a), and consistent with the formation of higher order oligomers. (PDF 1825 kb)
Additional file 8: Figure S8. Comparison of ^15^N-BEST-TROSY spectra of the LITAF Δ114–139 and N-Helix constructs. The N-helix construct can be assumed to adopt a wild type (WT)-like fold as its sequence only differs in mutations that remove the amphipathic nature of the helix, whilst maintaining a helical secondary structure. The comparison of amide chemical shifts of LITAF N-helix with the truncated Δ114–139 construct reveals very few changes in the chemical environment that the structured LITAF domain is exposed to, with just a small number of shifts seen in residues that are close to the predicted helix/truncation point (labelled residues). This strongly suggests that, while the truncated LITAF Δ114–139 construct does not contain the predicted helix of the WT protein, the data gleaned from this construct is comparable to a pseudo-WT construct. (PDF 632 kb)
Additional file 9: Figure S9. (a) The secondary structure boundaries of the LITAF Δ114–139 construct as defined by the TALOS+ analysis of the backbone assignment. Areas of β-sheet are shown with blue arrows. (b) The heteronuclear NOE (hetNOE) analysis of the LITAF Δ114–139 construct. When the HetNOE values are positive and close to 1, residues have a reduced motion on the picosecond time scale and are likely to be part of defined secondary structure motifs. The unstructured N-terminus has negative peaks suggesting increased flexibility on the picosecond time scale, with the exception of a small region between residues Ser22 and Tyr33. (PDF 332 kb)
Additional file 10: Figure S10.
^15^N-BEST-TROSY spectra of LITAF Δ114–139 wild type (WT; black) overlaid with phosphoethanolamine (PE, pink), phosphoglycerol (PG, yellow), phosphocholine (PC, blue), phosphoserine (PS, orange), and inositolhexakisphosphate (IP_6_, red), respectively. These data show that, while LITAF Δ114–139 WT does not interact with PG, PC, PS or IP_6_, discrete interactions between specific residues located in the C-terminal LITAF domain and PE head groups were observed (top right panel). (PDF 378 kb)
Additional file 11: Figure S11. (a) The weighted chemical shift perturbations that occur in the LITAF Δ114–139 wild type construct in the presence of phosphoethanolamine (PE) are shown. In summary, PE interacts with specific residues in the C-terminal LITAF domain. (b) The weighted chemical shift perturbations seen in the LITAF Δ114–139 construct harbouring the CMT1C-associated pathogenic mutation, V144M, compared to wild type. This mutation not only causes chemical shifts in the local vicinity of the substitution site (the mutated residue is shown in grey) but also affects residues in the other β-sheet region on the N-terminal side of the LITAF domain. (PDF 266 kb)
Additional file 12: Figure S12. (a) The position of disease-associated residues mutated in CMT1C are shown in magenta on the structural model of the human LITAF domain. Note that the residues mutated in Charcot-Marie-Tooth disease are in and around the region of the predicted hydrophobic helical membrane anchor. Valine 144 (mutated to Methionine in CMT1C and present in the LITAF Δ114–139 construct) is circled. (b) Flag-tagged LITAF WT and LITAF V144M were transiently expressed in HeLa cells and visualised by confocal immunofluorescence microscopy. Both constructs targeted to punctate structures consistent with endosomes. Nuclei were visualised with DAPI. Scale bar denotes 10 μm. (c) Western blot from membrane fractionation experiments from HeLa cells transiently expressing Flag-tagged LITAF WT or Flag-tagged LITAF V144M. Both constructs are found in the membrane pellets (P), as opposed to the soluble fraction (S). Calnexin and tubulin were used as integral membrane and soluble protein controls, respectively. (PDF 12512 kb)
Additional file 13: Figure S13.
^15^N-BEST-TROSY spectra of the LITAF Δ114–139 wild type (WT; dark blue, top panels) and LITAF Δ114–139 V144M (red, bottom panels) constructs overlaid with 1:250 and 1:1000 PE mixtures, respectively. These data show that whilst both the WT and V144M constructs interact with PE head groups, the V144M construct becomes insoluble at the higher PE concentrations. (PDF 673 kb)
Additional file 14: Individual data values from three biological replicates, each consisting of 10–13 technical replicates, used to quantitate the degree of colocalisation between HA-LITAF constructs and wheat germ agglutinin as illustrated in Fig. 7d. (XLSX 11 kb)
