# Supplementary material for: The topology, structure and PE interaction of LITAF underpin a Charcot-Marie-Tooth disease type 1C
Source: BMC Biol. 2016 Dec 7;14:109. doi: 10.1186/s12915-016-0332-8 (PMC5142333; doi:10.1186/s12915-016-0332-8)
Supplement: Additional file 1: Figure S1. — LITAF domains are conserved across the eukaryotes. Coulson plot showing the distribution of LITAF domain-containing proteins across the eukaryotes. Filled circles indicate the presence of a gene encoding a protein with an identifiable LITAF domain. Unfilled circles indicate taxa where no LITAF domain proteins could be identified. Numbers within circles indicate the number of distinct genes encoding proteins bearing LITAF domains. (PDF 77 kb) [file 12915_2016_332_MOESM1_ESM.pdf]

Figure S1

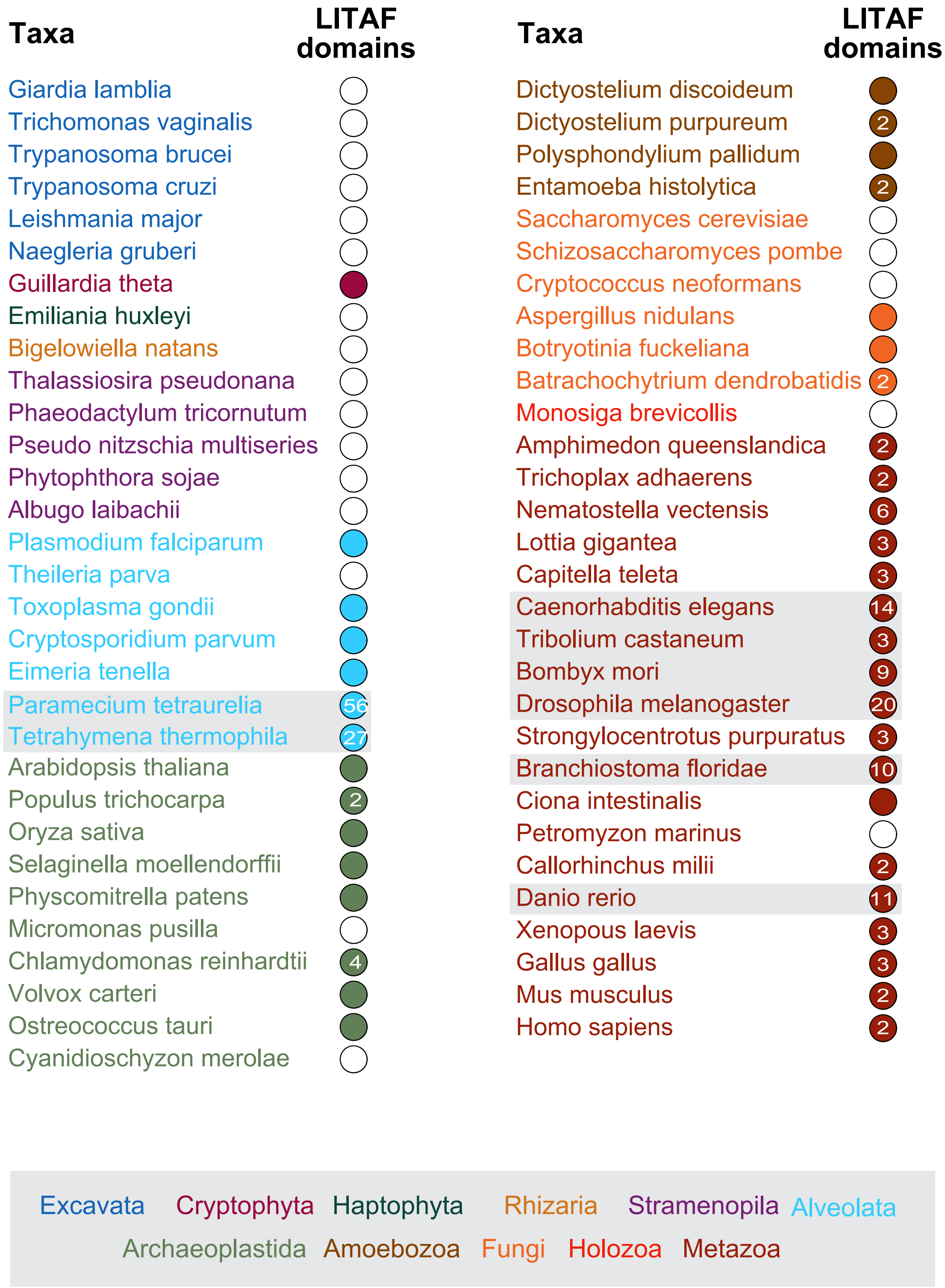
Taxa
LITAF domains
Giardia lamblia
Trichomonas vaginalis
Trypanosoma brucei
Trypanosoma cruzi
Leishmania major
Naegleria gruberi
Guillardia theta
Emiliania huxleyi
Bigelowiella natans
Thalassiosira pseudonana
Phaeodactylum tricornutum
Pseudo nitzschia multiseries
Phytophthora sojae
Albugo laibachii
Plasmodium falciparum
Theileria parva
Toxoplasma gondii
Cryptosporidium parvum
Eimeria tenella
Paramecium tetraurelia 56
Tetrahymena thermophila 27
Arabidopsis thaliana
Populus trichocarpa 2
Oryza sativa
Selaginella moellendorffii
Physcomitrella patens
Micromonas pusilla
Chlamydomonas reinhardtii 4
Volvox carteri
Ostreococcus tauri
Cyanidioschyzon merolae
Taxa
LITAF domains
Dictyostelium discoideum
Dictyostelium purpureum 2
Polysphondylium pallidum
Entamoeba histolytica 2
Saccharomyces cerevisiae
Schizosaccharomyces pombe
Cryptococcus neoformans
Aspergillus nidulans
Botryotinia fuckeliana
Batrachochytrium dendrobatidis 2
Monosiga brevicollis
Amphimedon queenslandica 2
Trichoplax adhaerens 2
Nematostella vectensis 6
Lottia gigantea 3
Capitella teleta 3
Caenorhabditis elegans 14
Tribolium castaneum 3
Bombyx mori 9
Drosophila melanogaster 20
Strongylocentrotus purpuratus 3
Branchiostoma floridae 10
Ciona intestinalis
Petromyzon marinus
Callorhinchus milii 2
Danio rerio 11
Xenopous laevis 3
Gallus gallus 3
Mus musculus 2
Homo sapiens 2
Excavata
Cryptophyta
Haptophyta
Rhizaria
Stramenopila
Alveolata
Archaeoplastida
Amoebozoa
Fungi
Holozoa
Metazoa
