# Supplementary figures and images for: The topology, structure and PE interaction of LITAF underpin a Charcot-Marie-Tooth disease type 1C
Source: BMC Biol. 2016 Dec 7;14:109. doi: 10.1186/s12915-016-0332-8 (PMC5142333; doi:10.1186/s12915-016-0332-8)

Figure S2

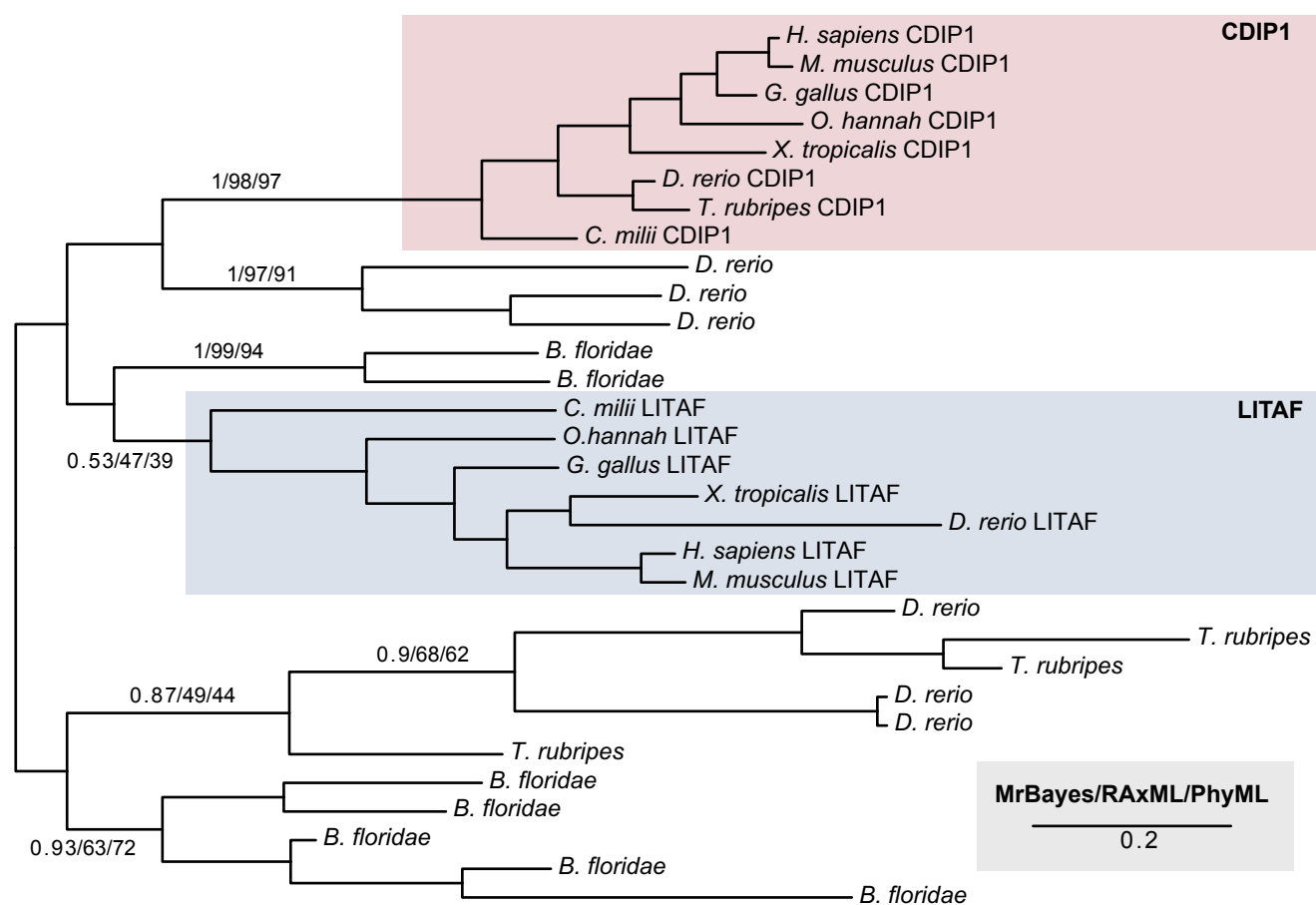

Supplement: Additional file 2: Figure S2. — Phylogenetic reconstruction of the evolution of LITAF domain proteins within the chordates. The best Bayesian topology is shown (MrBayes) with branch support for key nodes indicated according to the key. (PDF 33 kb) [file 12915_2016_332_MOESM2_ESM.pdf]

Figure S3

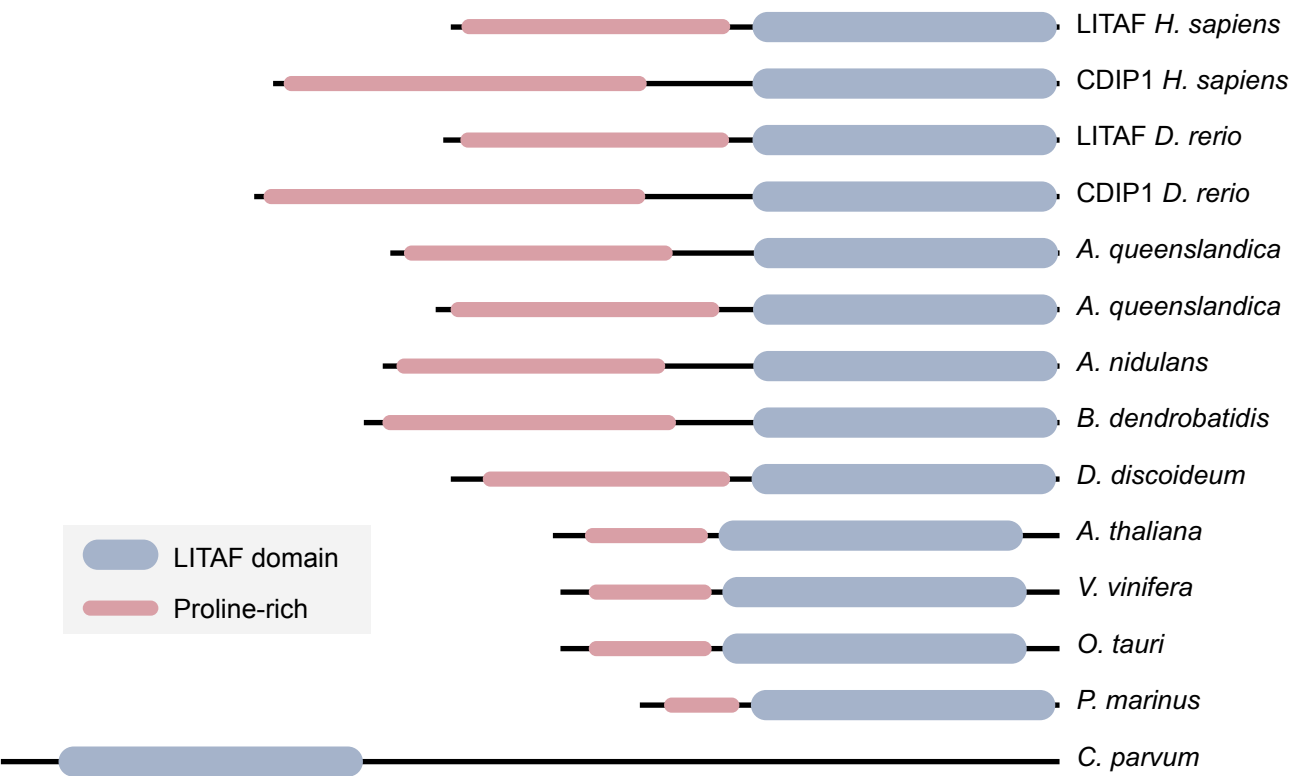

Supplement: Additional file 3: Figure S3. — Predicted domain organisation of representative LITAF domain proteins from across the eukaryotes. (PDF 36 kb) [file 12915_2016_332_MOESM3_ESM.pdf]

Figure S5

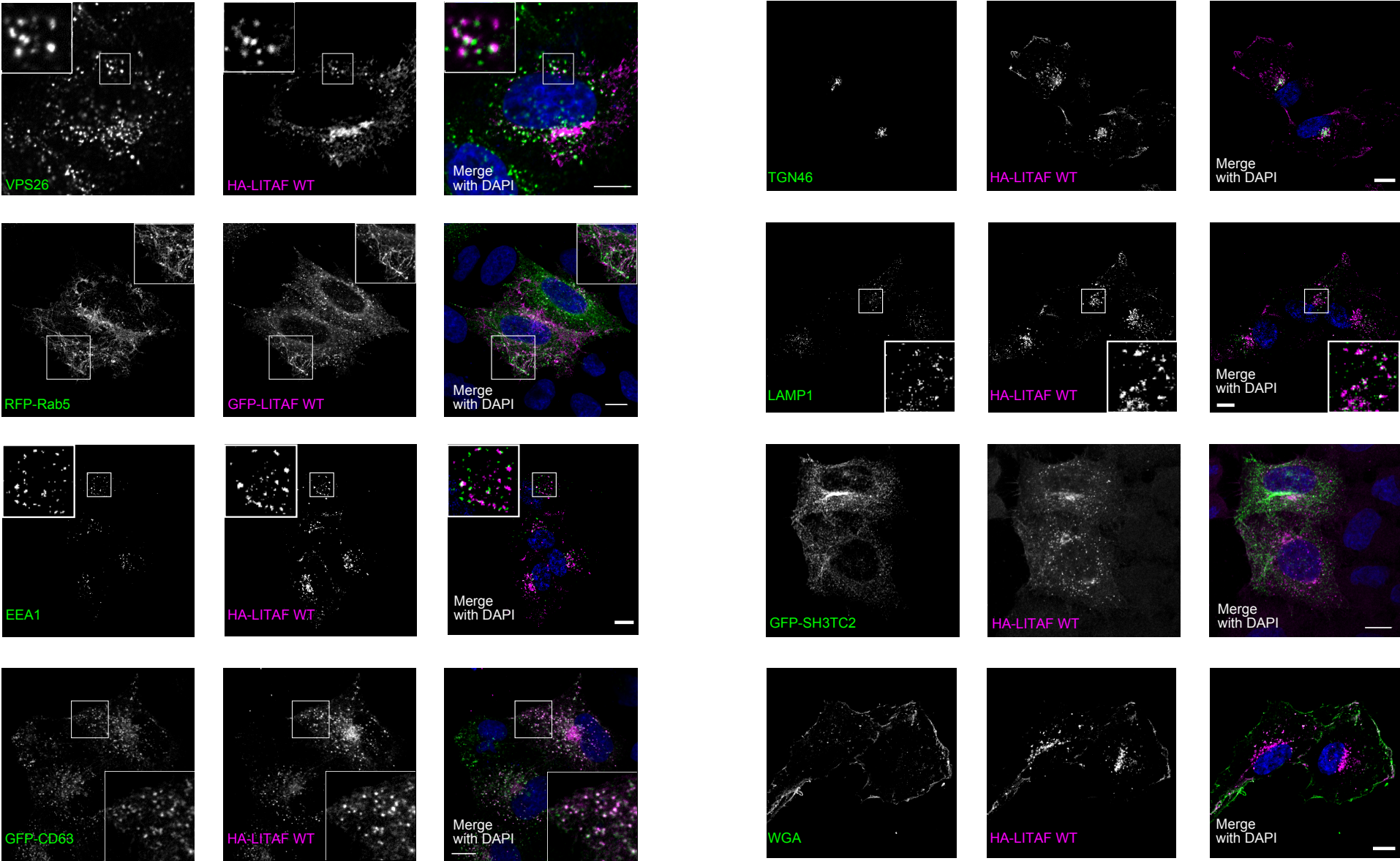

Supplement: Additional file 5: Figure S5. — Intracellular localisation of LITAF. Colocalisation immunofluorescence studies of wild type (WT) LITAF constructs stably expressed in retinal epithelial cells (RPE) by confocal microscopy. Epitope-tagged LITAF WT targeted to punctate structures with partial colocalisation with markers of early endosomes (EEA1, Rab5), late endosomes/lysosomes (CD63, LAMP1) and with the retromer component, VPS26. Minimal colocalisation of HA-LITAF WT with the trans Golgi network marker (TGN46) or plasma membrane marker, wheat germ agglutinin, was observed. Scale bar denotes 10 μm. (PDF 3672 kb) [file 12915_2016_332_MOESM5_ESM.pdf]

Figure S6

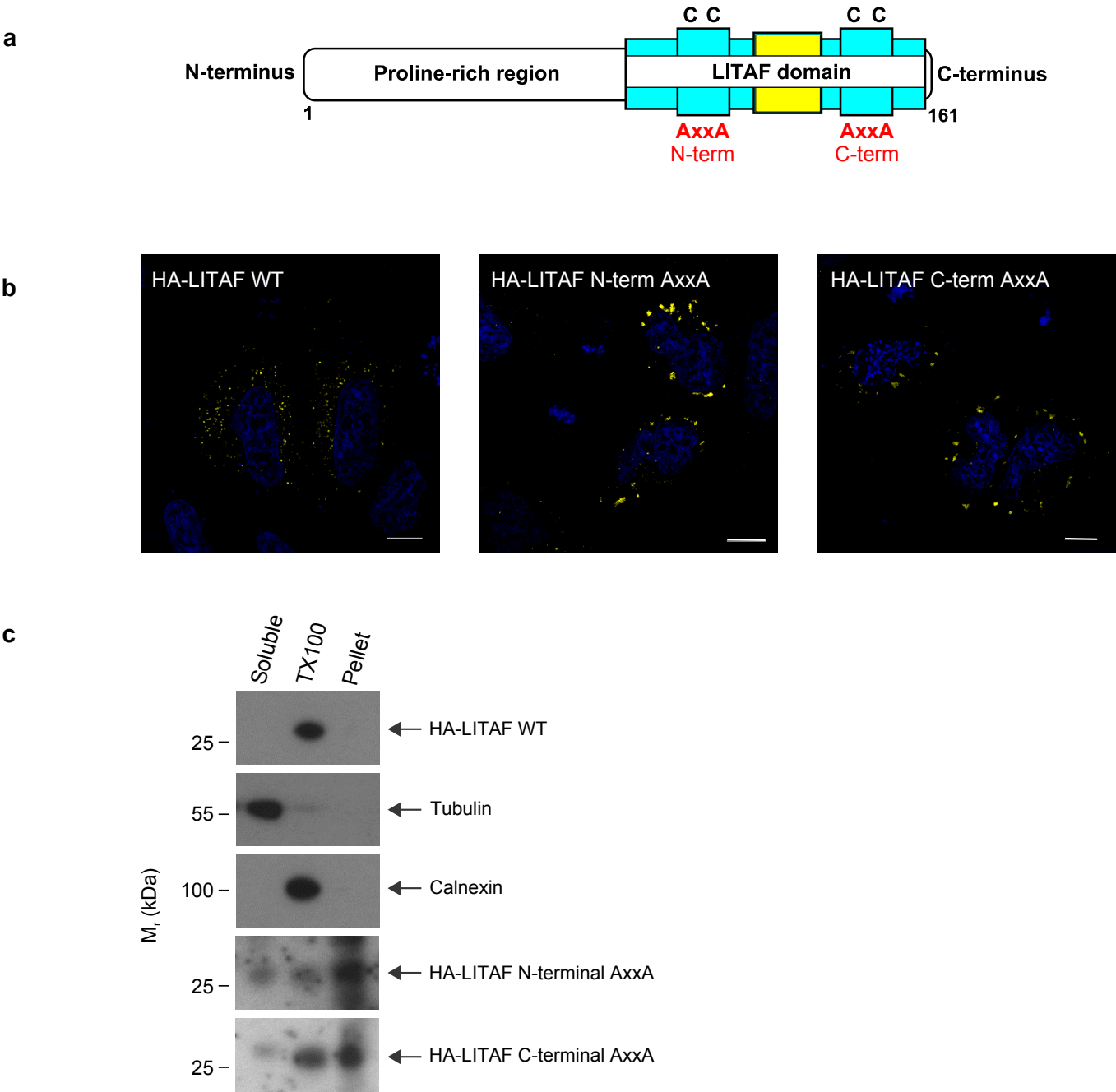

Supplement: Additional file 6: Figure S6. — (a) Schematic diagram illustrating the position of the conserved cysteine residues mutated to alanine in the N-term AxxA and C-term AxxA HA-LITAF constructs. (b) HA-LITAF constructs were transiently expressed in HeLa cells and visualised by immunofluorescence confocal microscopy. In contrast to the wild type LITAF, which targeted multiple punctate structures consistent with endosomes (HA-LITAF WT, left panel), the LITAF constructs in which the N-terminal or C-terminal conserved cysteine pairs were mutated to alanine (HA-LITAF N-term AxxA and HA-LITAF C-term AxxA) appeared in large puncta, consistent with them being proteins prone to aggregation. Nuclei were stained with DAPI. Scale bars denote 10 μm. (c) Membrane fractionation from HeLa cells transiently expressing HA-LITAF constructs. A soluble fraction (Soluble) was obtained following high speed centrifugation. The resulting membrane pellet was further incubated with 1% TX100 in order to extract soluble membrane proteins (TX100) from insoluble material (Pellet). Calnexin and tubulin were used for control purposes as examples of integral membrane and soluble proteins, respectively. While HA-LITAF WT was found in the TX100 solubilised membrane fraction similarly to calnexin, HA-LITAF construct harbouring mutations in either the N-terminal or C-terminal conserved cysteine pairs within the LITAF domain were found predominantly in the TX100 insoluble pellet (P), consistent with protein aggregation. Note that longer exposure times were required for the cysteine mutant samples as the expression level of these mutated proteins was consistently lower compared to wild type. (PDF 246 kb) [file 12915_2016_332_MOESM6_ESM.pdf]

**Figure S7**

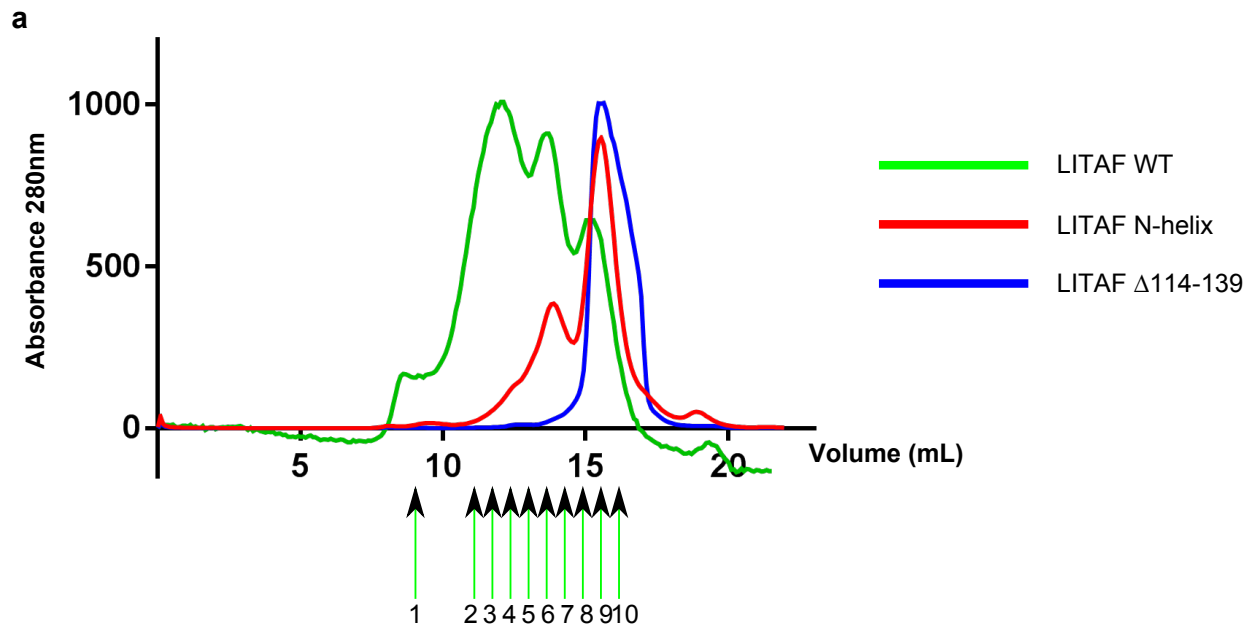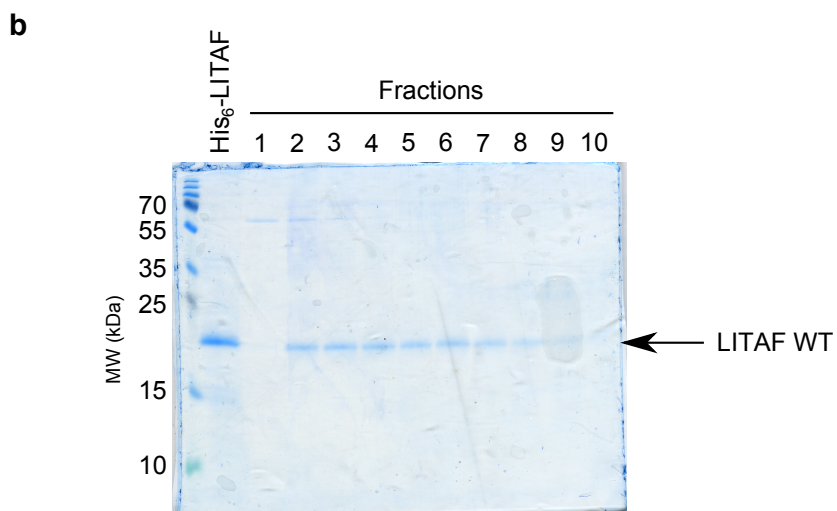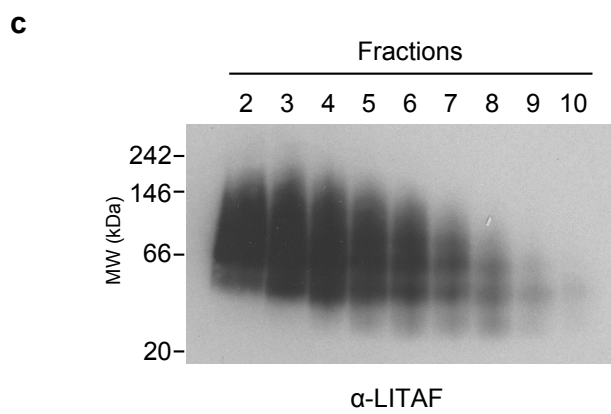

Supplement: Additional file 7: Figure S7. — (a) Representative traces of recombinantly-expressed LITAF wild type (WT; green), LITAF N-helix (red) and LITAF Δ114–139 (blue) subjected to size exclusion chromatography (SEC) using a Superdex 200 10/300 GL column (GE Healthcare Life Sciences). The broad peak on the SEC column corresponding to LITAF WT indicated the presence of higher order oligomers. (b) Fractions from the size exclusion chromatography of LITAF WT (arrows labelled 1–10 in (a)) were separated by SDS-PAGE and visualised with InstantBlue (Expedeon). A protein band corresponding to LITAF WT was present in fractions 2–10. (c) Western blot of fractions 2–10 from the size exclusion chromatography of LITAF WT separated by native gel electrophoresis before transfer to a nitrocellulose membrane and immunoblotted for LITAF. Note that LITAF WT exists as larger molecular weight species in the earlier fractions compared to the later fractions, consistent with the size exclusion chromatography trace seen in (a), and consistent with the formation of higher order oligomers. (PDF 1825 kb) [file 12915_2016_332_MOESM7_ESM.pdf]

Figure S8

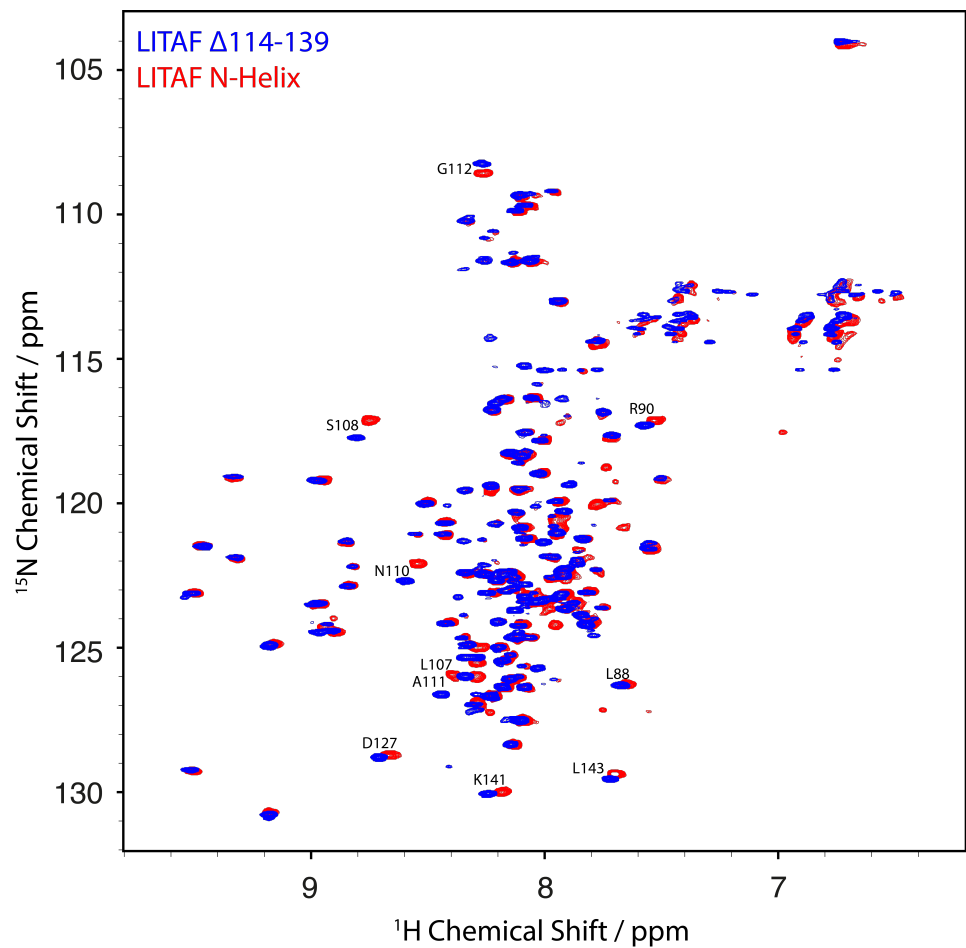

Supplement: Additional file 8: Figure S8. — Comparison of 15N-BEST-TROSY spectra of the LITAF Δ114–139 and N-Helix constructs. The N-helix construct can be assumed to adopt a wild type (WT)-like fold as its sequence only differs in mutations that remove the amphipathic nature of the helix, whilst maintaining a helical secondary structure. The comparison of amide chemical shifts of LITAF N-helix with the truncated Δ114–139 construct reveals very few changes in the chemical environment that the structured LITAF domain is exposed to, with just a small number of shifts seen in residues that are close to the predicted helix/truncation point (labelled residues). This strongly suggests that, while the truncated LITAF Δ114–139 construct does not contain the predicted helix of the WT protein, the data gleaned from this construct is comparable to a pseudo-WT construct. (PDF 632 kb) [file 12915_2016_332_MOESM8_ESM.pdf]

Figure S9

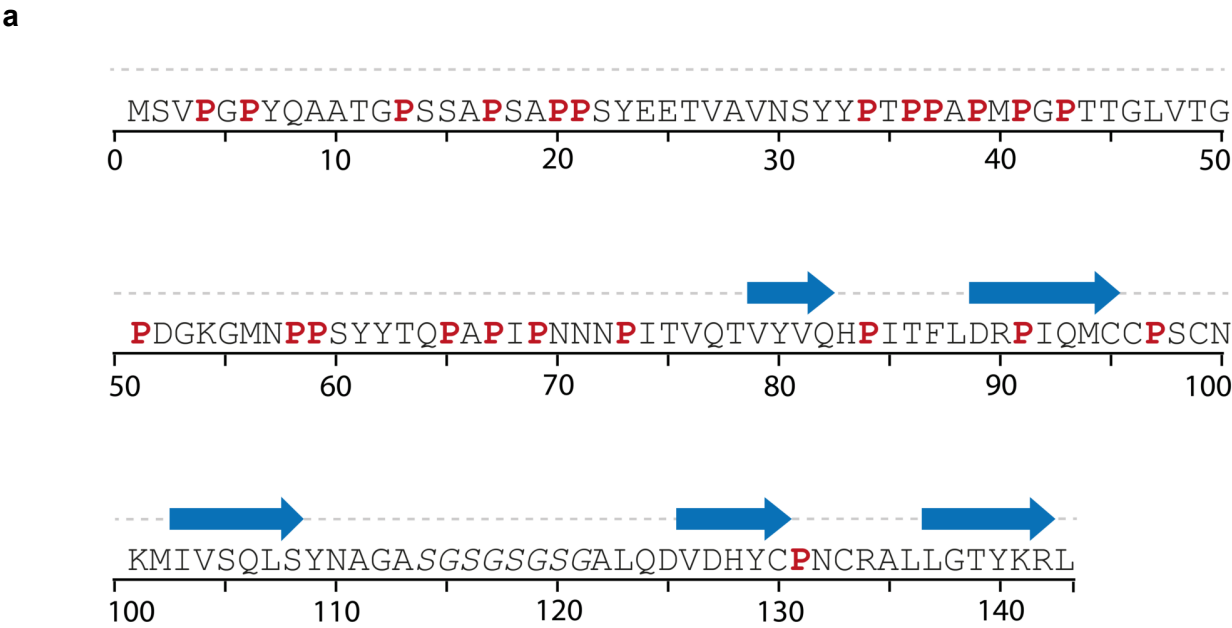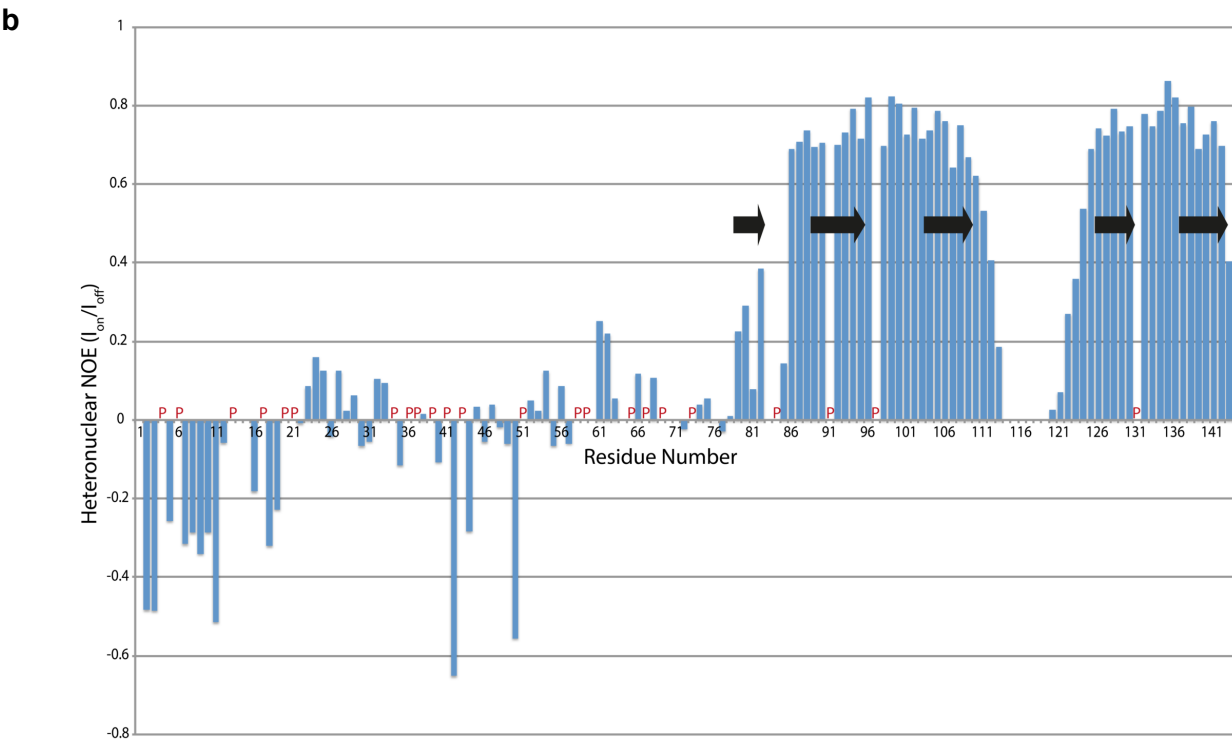

Supplement: Additional file 9: Figure S9. — (a) The secondary structure boundaries of the LITAF Δ114–139 construct as defined by the TALOS+ analysis of the backbone assignment. Areas of β-sheet are shown with blue arrows. (b) The heteronuclear NOE (hetNOE) analysis of the LITAF Δ114–139 construct. When the HetNOE values are positive and close to 1, residues have a reduced motion on the picosecond time scale and are likely to be part of defined secondary structure motifs. The unstructured N-terminus has negative peaks suggesting increased flexibility on the picosecond time scale, with the exception of a small region between residues Ser22 and Tyr33. (PDF 332 kb) [file 12915_2016_332_MOESM9_ESM.pdf]

**Figure S10**

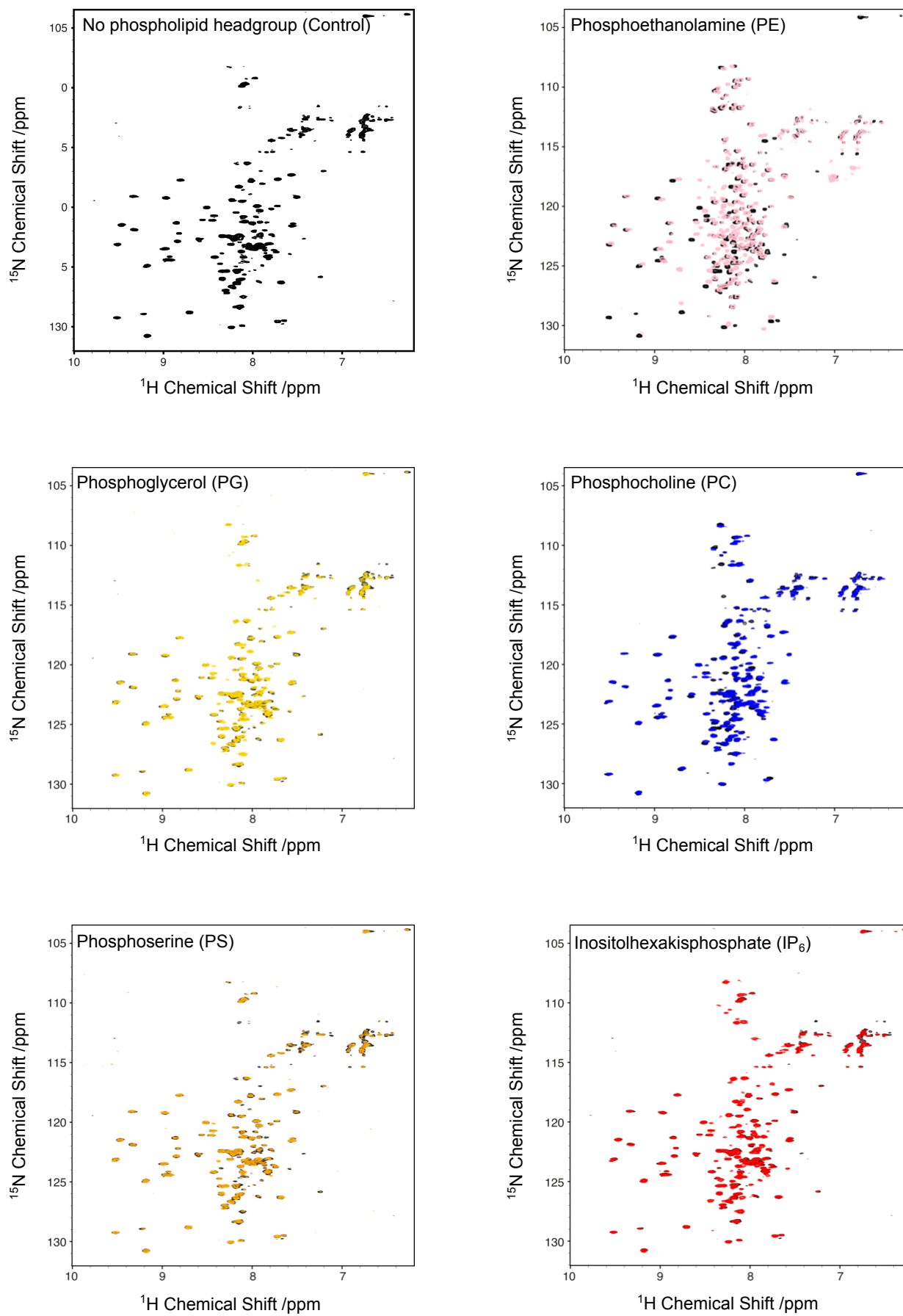

Supplement: Additional file 10: Figure S10. — 15N-BEST-TROSY spectra of LITAF Δ114–139 wild type (WT; black) overlaid with phosphoethanolamine (PE, pink), phosphoglycerol (PG, yellow), phosphocholine (PC, blue), phosphoserine (PS, orange), and inositolhexakisphosphate (IP6, red), respectively. These data show that, while LITAF Δ114–139 WT does not interact with PG, PC, PS or IP6, discrete interactions between specific residues located in the C-terminal LITAF domain and PE head groups were observed (top right panel). (PDF 378 kb) [file 12915_2016_332_MOESM10_ESM.pdf]

Figure S11

**a**

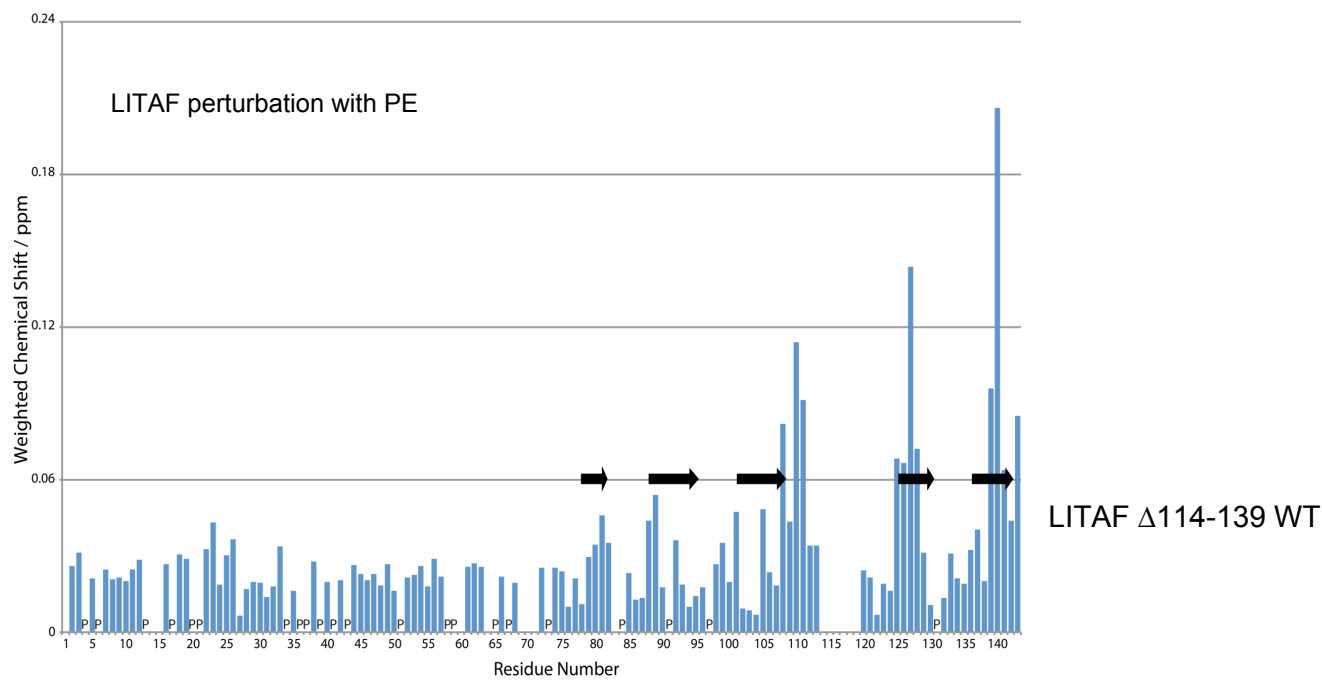

**b**

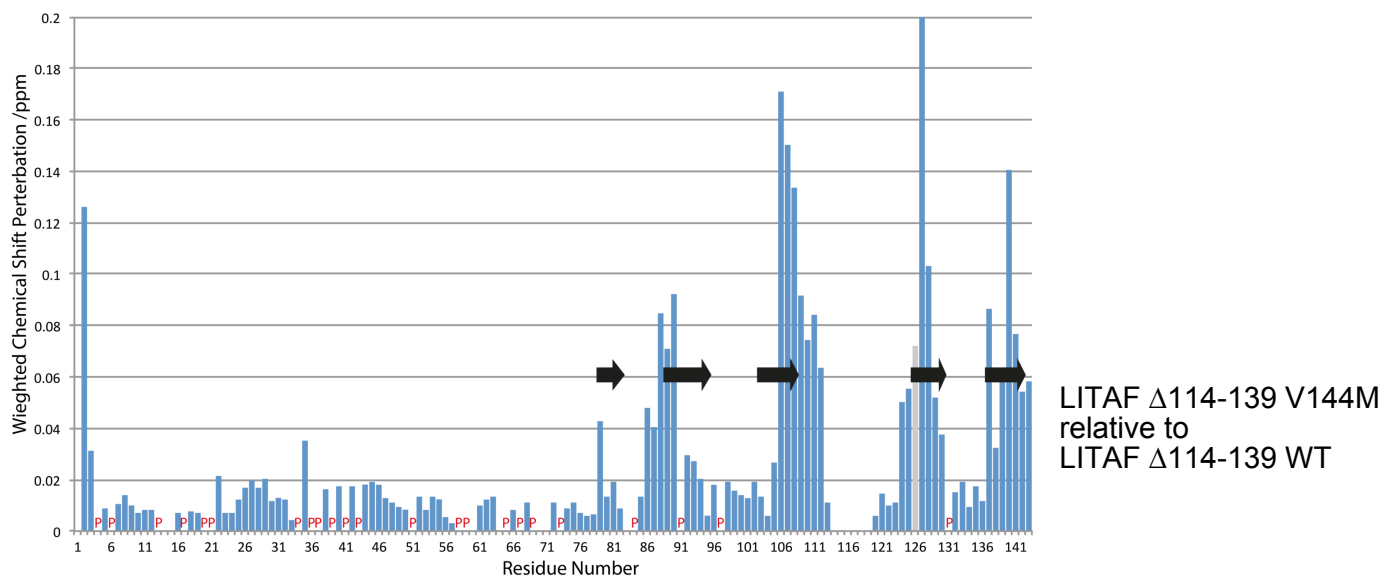

Supplement: Additional file 11: Figure S11. — (a) The weighted chemical shift perturbations that occur in the LITAF Δ114–139 wild type construct in the presence of phosphoethanolamine (PE) are shown. In summary, PE interacts with specific residues in the C-terminal LITAF domain. (b) The weighted chemical shift perturbations seen in the LITAF Δ114–139 construct harbouring the CMT1C-associated pathogenic mutation, V144M, compared to wild type. This mutation not only causes chemical shifts in the local vicinity of the substitution site (the mutated residue is shown in grey) but also affects residues in the other β-sheet region on the N-terminal side of the LITAF domain. (PDF 266 kb) [file 12915_2016_332_MOESM11_ESM.pdf]

Figure S12

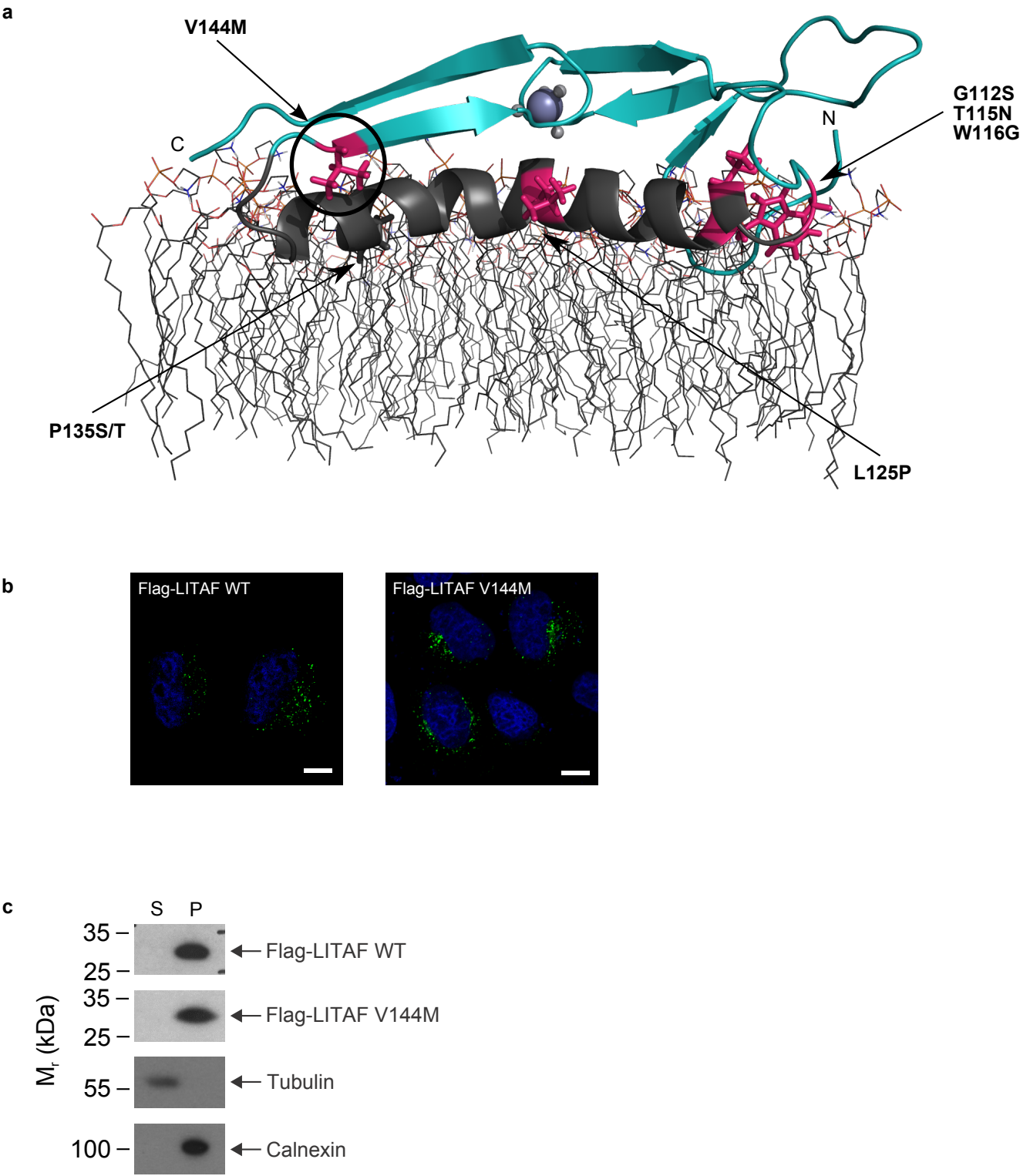

Supplement: Additional file 12: Figure S12. — (a) The position of disease-associated residues mutated in CMT1C are shown in magenta on the structural model of the human LITAF domain. Note that the residues mutated in Charcot-Marie-Tooth disease are in and around the region of the predicted hydrophobic helical membrane anchor. Valine 144 (mutated to Methionine in CMT1C and present in the LITAF Δ114–139 construct) is circled. (b) Flag-tagged LITAF WT and LITAF V144M were transiently expressed in HeLa cells and visualised by confocal immunofluorescence microscopy. Both constructs targeted to punctate structures consistent with endosomes. Nuclei were visualised with DAPI. Scale bar denotes 10 μm. (c) Western blot from membrane fractionation experiments from HeLa cells transiently expressing Flag-tagged LITAF WT or Flag-tagged LITAF V144M. Both constructs are found in the membrane pellets (P), as opposed to the soluble fraction (S). Calnexin and tubulin were used as integral membrane and soluble protein controls, respectively. (PDF 12512 kb) [file 12915_2016_332_MOESM12_ESM.pdf]

Figure S13

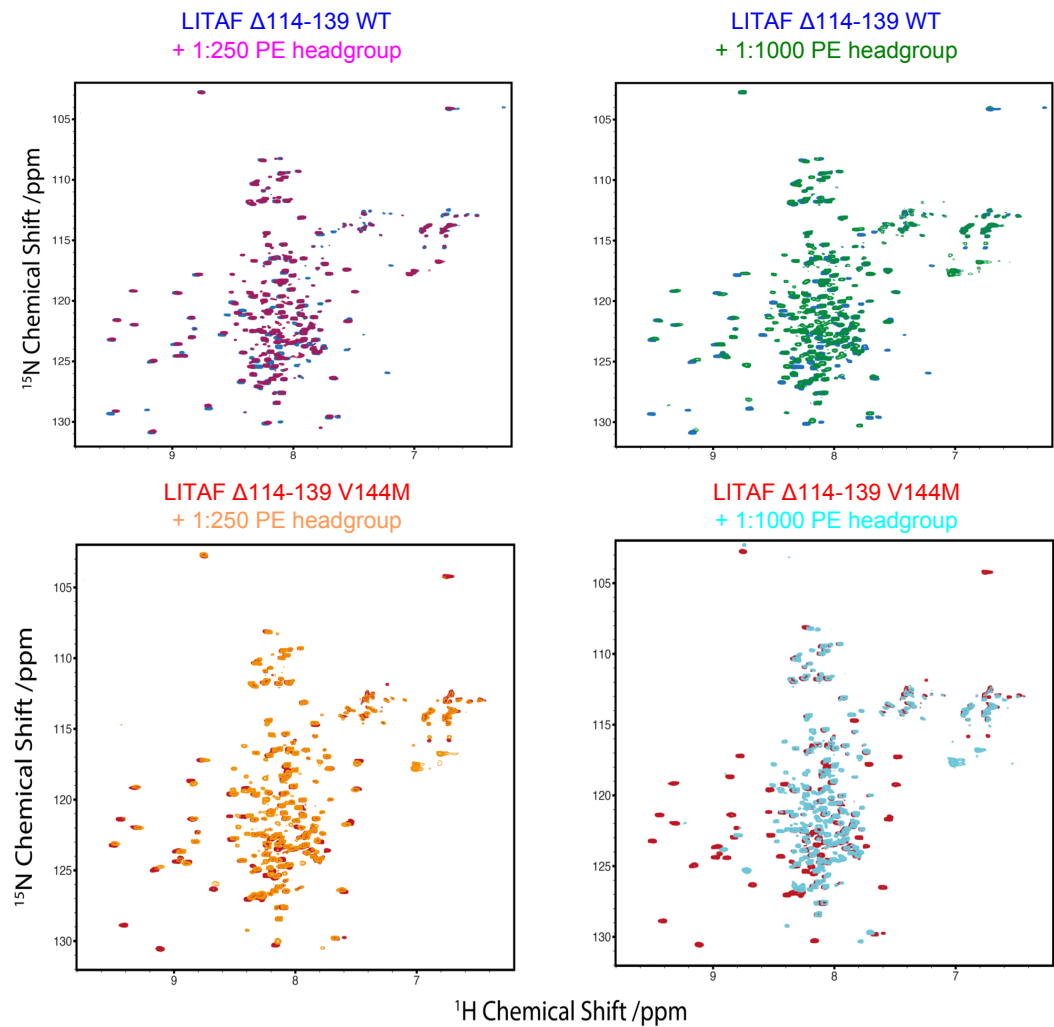

Supplement: Additional file 13: Figure S13. — 15N-BEST-TROSY spectra of the LITAF Δ114–139 wild type (WT; dark blue, top panels) and LITAF Δ114–139 V144M (red, bottom panels) constructs overlaid with 1:250 and 1:1000 PE mixtures, respectively. These data show that whilst both the WT and V144M constructs interact with PE head groups, the V144M construct becomes insoluble at the higher PE concentrations. (PDF 673 kb) [file 12915_2016_332_MOESM13_ESM.pdf]
