# Supplementary material for: The topology, structure and PE interaction of LITAF underpin a Charcot-Marie-Tooth disease type 1C
Source: BMC Biol. 2016 Dec 7;14:109. doi: 10.1186/s12915-016-0332-8 (PMC5142333; doi:10.1186/s12915-016-0332-8)

Figure S4

a

1 M S V P G P Y Q A A T G P S S A P S A P P S Y E E T

27 V A V N S Y Y P T P P A P M P G P T T G L V T G P D

53 G K G M N P P S Y Y T Q P A P I P N N N P I T V Q T

79 V Y V Q H P I T F L D R P I Q M C C P S C N K M I V

105 S Q L S Y N A G A L T W L S C G S L C L L G C I A G

131 C C F I P F C V D A L Q D V D H Y C P N C R A L L G

157 T Y K R L

b

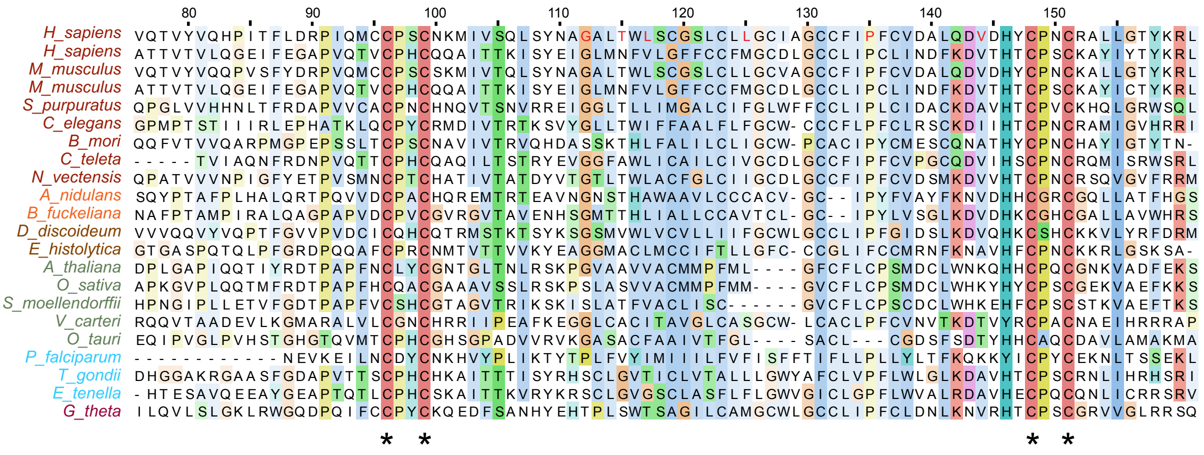

c

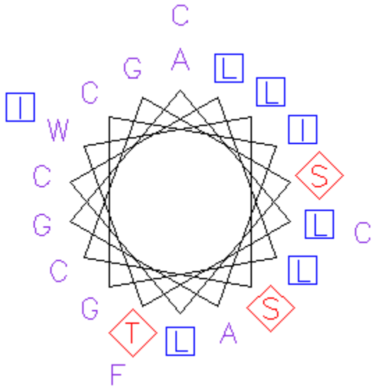

d

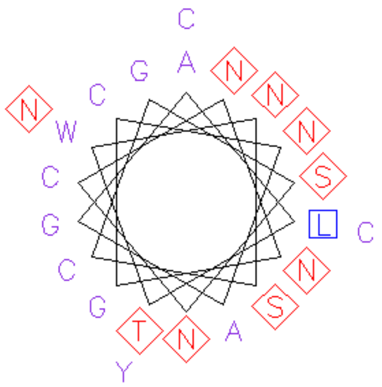

Supplement: Additional file 4: Figure S4. — Sequence of human LITAF and alignment of representative eukaryote LITAF domains. (a) Amino acid sequence of human LITAF. Residues comprising the C-terminal LITAF domain are outlined in red. The two PPSY motifs that are predicted to interact with WW domains, and the PSAP motif that can associate with the UEV domain of TSG101 that are present in the N-terminal proline-rich region are outlined by blue and green boxes, respectively. (b) LITAF domain alignment constructed from representative taxa encompassing the diversity of eukaryotes. Residues are numbered according to the human LITAF sequence and coloured according to the Clustalx scheme as implemented in the Jalview program to highlight conserved amino acid properties. Shading indicates the degree of conservation. The conserved cysteine residues are coloured red and denoted with asterisks. (c) Helical wheel alignment of residues 113–134 of LITAF, predicted to form a helix by Jpred4 [86], to illustrate the predicted clustering of hydrophobic residues on one face of the helix, compatible with an amphipathic helix. (d) Helical wheel alignment of residues 113–134 of LITAF, with the hydrophobic residues on one face of the helix substituted with asparagine (N), as found in the soluble LITAF N-helix construct. (PDF 1131 kb) [file 12915_2016_332_MOESM4_ESM.pdf]
